# Deficiency of Leucine‐Rich Repeat Containing G Protein‐Coupled Receptor 4 in Pancreas Reduces β Cell Mass

**DOI:** 10.1002/advs.202508858

**Published:** 2025-08-14

**Authors:** Chao Luo, Yifan Feng, Jiajie Min, Yan Zhao, Ziming Zhu, Lijun Sun, Hao Yin, Yue Yin, Weizhen Zhang

**Affiliations:** ^1^ Department of Physiology and Pathophysiology School of Basic Medical Sciences State Key Laboratory of Vascular Homeostasis and Remodeling Peking University Beijing 100191 China; ^2^ Department of Pharmacology School of Basic Medical Sciences State Key Laboratory of Vascular Homeostasis and Remodeling Peking University Beijing 100191 China; ^3^ Organ Transplant Center Shanghai Changzheng Hospital Second Affiliated Hospital of Naval Medical University Shanghai 200003 China

**Keywords:** apoptosis, glucose metabolism, islet β cell mass, leucine‐rich repeat containing G protein‐coupled receptor 4, proliferation

## Abstract

Although leucine‐rich repeat‐containing G protein‐coupled receptor 4 (LGR4) is abundantly expressed in the pancreas, it is currently unknown whether LGR4 impacts pancreatic endocrine cells. Here, a critical role of LGR4 is demonstrated in islet β cell mass using a group of transgenic mice with LGR4 deficiency. Knock‐out of *Lgr4* in the pancreas and islet β cells significantly reduced islet β cell mass, and subsequently impaired glucose metabolism upon the challenge of a high‐fat diet. Deficiency of LGR4 in these mice or in cultured INS‐1 cells showed a significant reduction in islet β cell proliferation measured by Ki‐67, EdU, and CCK‐8 assay. Increase of islet β cell proliferation induced by Rspondin‐LGR4 signaling occurred via Wnt‐β‐catenin‐*Ccnd1* axis. In addition, the deficiency of LGR4 in islet β cells significantly increased apoptosis. Inhibition of RANKL‐RANK signaling by the TRAF‐STOP inhibitor significantly attenuated apoptosis of cultured INS‐1 cells induced by deficiency of LGR4. Overall, this work shows that deficiency of LGR4 reduces islet β cell mass via suppression of proliferation and concurrent increase of apoptosis. LGR4 in pancreatic islets is thus critical for the control of glucose homeostasis.

## Graphical abstract

In islet β cells, LGR4 binds with Rspondin to activate the Wnt‐β‐catenin‐*Ccnd1* axis, leading to a subsequent increase in cell proliferation. On the other hand, LGR4 also binds with RANKL, thus suppressing the phosphorylation of p44/p42 MAPK and p65 NF‐κB and subsequent cell apoptosis. LGR4 is thus a potential therapeutic target to regulate the flexibility of islet β cells and treat diabetes.

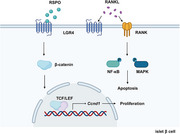

## 1 Introduction

As the most extensive targets of drug development,^[^ ^1^ ^]^ G protein‐coupled receptors (GPCRs) including glucagon‐like peptide 1 receptor (GLP1R),^[^ ^2^ ^]^ G protein‐coupled receptor 119 (GPR119),^[^ ^3^ ^]^ and free fatty acid receptor 1 (FFAR1)^[^ ^4^ ^]^ have already been shown promising effects on the intervention of metabolic disorders. By screening the GPCRs relevant to glucose and lipid metabolism in publicly available databases and literature,^[^ ^5^ ^]^ we have identified a special GPCR, leucine‐rich repeat‐containing G protein‐coupled receptor 4 (LGR4), as an interesting target.

Despite being a GPCR, LGR4 does not canonically function via G proteins or β‐arrestin. Instead, LGR4 works through binding with its endogenous ligands: Rspondin1‐4 (RSPO1‐4) to promote the ubiquitination and degradation of zinc and ring finger 3 (ZNRF3)/ring finger protein 43 (RNF43). This decreases the ubiquitination of Frizzled and subsequently enhances the activation of the Wnt‐β‐catenin signaling pathway.^[^ ^6^, ^7^, ^8^, ^9^, ^10^ ^]^ Besides, activation of classical G protein‐coupled signaling cascade by newly identified ligands such as receptor activator of nuclear factor kappa Β ligand (RANKL)^[^ ^11^ ^]^ and Nidogen‐2^[^ ^12^ ^]^ has also been reported. These observations suggest the functional diversity of LGR4. For example, LGR4 has been demonstrated to be critical for the control of energy metabolism. Its expression and physiological functions in multiple metabolic organs and tissues such as hypothalamus,^[^ ^13^, ^14^ ^]^ liver,^[^ ^15^, ^16^ ^]^ intestine^[^ ^17^ ^]^ and white adipose tissue^[^ ^18^ ^]^ have been reported. However, whether LGR4 plays a role in pancreatic endocrine cells, which are critical for glucose metabolism, remains unclear.

Here, we reported that LGR4 deficiency reduced islet β cell mass, which rendered mice vulnerable to dysfunction of glucose metabolism induced by a high‐fat diet (HFD). Using pancreas‐specific *Lgr4* knock‐out mice (*Lgr4 flox/flox, Pdx1‐Cre+/‐*: *Lgr4^pko^* mice), we demonstrated that deficiency of LGR4 in the pancreas decreased islet β cell mass but not that of α or δ cells. Further, we demonstrated that LGR4 participated in the regulation of islet β cells in the post‐development stages using inducible islet β cell‐specific *Lgr4* knock‐out mice (*Lgr4 flox/flox, Pdx1‐CreER+/‐*: *iLgr4^pko^* mice and *Lgr4 flox/flox, Ins2‐CreER+/‐*: *iLgr4^bko^* mice). As for the mechanism, we found that LGR4 promoted islet β cell proliferation via binding with RSPO and activating the Wnt‐β‐catenin‐*Ccnd1* axis. Concurrently, LGR4 suppressed islet β cell apoptosis via competing with RANKL for binding with RANK, leading to inhibition of the phosphorylation of p44/p42 MAPK and p65 NF‐κB.

## 2 Results

### 2.1 Deficiency of Islet LGR4 is related with Metabolic Disorders

In human beings, at least 13 mutations in the *LGR4* gene locus are correlated with metabolic disorders (Table S1, Supporting Information), indicating a close relation between LGR4 and metabolism. Interestingly, the mRNA level of *LGR4* in the pancreas is the most abundant among all organs and tissues analyzed in human beings (Figure S1A, Supporting Information). In the pancreas, LGR4 is mainly expressed in endocrine and ductal cells (Figure S1B, Supporting Information). Considering the critical role of islet endocrine cells in glucose and lipid metabolism, we analyzed the expression of *Lgr4* in different mouse models. In db/db (GSE107489)^[^ ^19^ ^]^ and our 16‐week‐ HFD induced obese mice, mRNA levels of islet *Lgr4* demonstrated either a significant decrement or a tendency to decrease (Figure S1C,D, Supporting Information). These results suggest that a deficiency of islet LGR4 is closely related to metabolic disorders.

### 2.2 Deficiency of Pancreatic LGR4 Reduces Islet β Cell Mass, Rendering Mice Vulnerable to Glucose Dysfunctions Induced by HFD

To explore the function of LGR4 in the pancreas and pancreatic endocrine cells, we first established a pancreas‐specific *Lgr4* knock‐out mouse model (*Lgr4 flox/flox, Pdx1‐Cre+/‐*: *Lgr4^pko^* mice) (Figure S1E–G, Supporting Information). Six to eight‐week‐old *Lgr4^pko^* mice and *Lgr4^fl/fl^* littermates fed a normal chow diet (NCD) for 16 weeks demonstrated a significant decrease in islet area (Figure S2B,C, Supporting Information) and β cell area (Figure S2D,E, Supporting Information), while areas of α and δ cells did not show significant differences (Figure S2F–I, Supporting Information). Functionally, *Lgr4^pko^* mice had higher random serum levels of glucagon (Figure S2J, Supporting Information) and lower random serum levels of insulin (Figure S2K, Supporting Information). C‐peptide levels (Figure S2L, Supporting Information) and insulin levels at 15 min after being stimulated by glucose (Figure S2N, Supporting Information) in these mice also decreased, although not significantly. Neither change were 16h fasting insulin levels (Figure S2M, Supporting Information) and insulin secretion of the islets when stimulated by low or high concentrations of glucose (Figure S2O, Supporting Information). These observations indicate that deficiency of pancreatic LGR4 reduces islet β cell mass, but has a negligible effect on glucose‐stimulated insulin secretion (GSIS) despite of the reduction of circulating insulin content in mice fed NCD.

**Figure 1:**
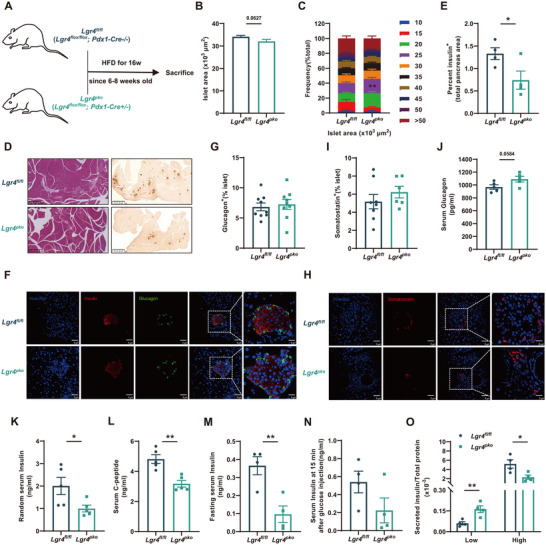
Deficiency of pancreas LGR4 decreases islet β cell mass and function. 6–8 week‐old *Lgr4^pko^* mice and *Lgr4^fl/fl^* littermates were fed with HFD for 16 weeks. Data are presented as mean±SEM. ^*^ *p*<0.05, ^**^ *p*<0.01 by t‐test. A) Schematic diagram of feeding strategy; B, C) Average islet area and distribution of islet area. *n* = 1176 and 409 islets isolated from *Lgr4^fl/fl^* and *Lgr4^pko^* mice respectively; D, E) Representative H&E, insulin immunochemistry staining, and percent insulin positive area per pancreas, *n* = 4; F, G) Representative insulin (Red) and glucagon (Green) immunofluorescence staining, and percentage of glucagon positive area per islet, *n* = 9; H, I) Representative somatostatin (Red) immunofluorescence staining, and percentage of somatostatin positive area per islet, *n* = 8 and 6 for *Lgr4^fl/fl^* and *Lgr4^pko^*; J) Random serum glucagon levels, *n* = 5; K) Random serum insulin levels, *n* = 5; L) Random serum C‐peptide levels, *n* = 5; M) 16 h fasting serum insulin levels, *n* = 4; N) Insulin levels at 15 min after glucose injection, *n* = 4; O) GSIS measured at low (2.8 mmol L^−1^) and high (25 mmol L^−1^) glucose levels, *n* = 4.

We next examined the effects of pancreatic LGR4 deficiency in *Lgr4^pko^* mice fed HFD. As shown in **Figure** **1**, *Lgr4^pko^* mice fed HFD showed a decrease in islet area (Figure 1B,C), islet β cell area (Figure 1D,E), random serum insulin levels (Figure 1K), and random serum C‐peptide levels (Figure 1L). Fasting serum insulin levels (Figure 1M) and insulin levels at 15 min after being stimulated by glucose (Figure 1N) also decreased. Interestingly, GSIS of these HFD‐feeding mice was significantly impaired (Figure 1O). Areas of islet α and δ cells remained unchanged (Figure 1F–I), while serum glucagon levels slightly increased (Figure 1J). All these results indicate that a deficiency of pancreatic LGR4 decreases islet β cell mass and GSIS in mice fed HFD.

**Figure 2:**
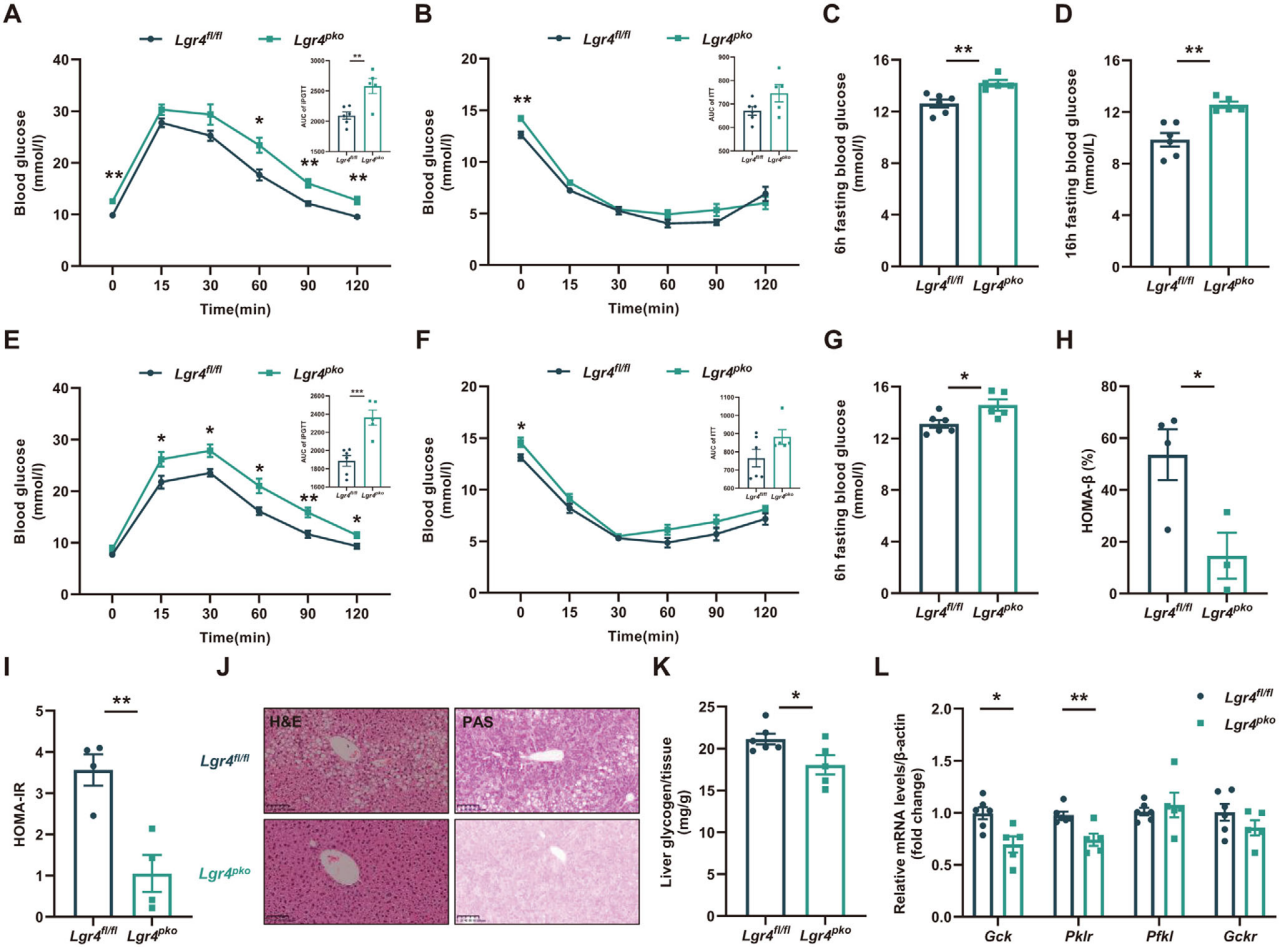
Deficiency of the pancreas LGR4 disrupts glucose metabolism upon the challenge of HFD. Data are presented as mean±SEM. ^*^ *p*<0.05, ^**^ *p*<0.01, ^***^ *p*<0.001 by t‐test. 6–8 week‐old *Lgr4^pko^* mice and *Lgr4^fl/fl^* littermates were fed with HFD for 8 weeks, *n* = 6 and 5 for *Lgr4^fl/fl^* and *Lgr4^pko^*. A) IPGTT and the area under the curve; B) ITT and the area under the curve; C) 6 h fasting blood glucose; D) 16h fasting blood glucose. 6–8 week‐old *Lgr4^pko^* mice and *Lgr4^fl/fl^* littermates were fed with HFD for 16 weeks, *n* = 6 and 5 for *Lgr4^fl/fl^* and *Lgr4^pko^*. E) IPGTT and the area under curve; F) ITT and the area under curve; G) 6 h fasting blood glucose; H) HOMA‐β values, *n* = 4; I) HOMA‐IR values, *n* = 4; J) Representative H&E and PAS staining of liver; K) Content of liver glycogen, normalized to the extracted tissue weight; L) Liver mRNA levels of genes relevant to glycolysis.

In *Lgr4^pko^* mice, the reduction in islet β cell mass and insulin secretion was associated with a significant impairment in glucose and lipid homeostasis. Glucose tolerance was significantly impaired in *Lgr4^pko^* mice fed NCD (Figure S2P, Supporting Information). While insulin tolerance (Figure S2Q, Supporting Information), 6 h fasting blood glucose (Figure S2R, Supporting Information), and 16 h fasting blood glucose (Figure S2S, Supporting Information) were not altered in transgenes fed NCD. The dysfunction of glucose metabolism was more obvious in *Lgr4^pko^* mice fed HFD. Glucose tolerance was markedly impaired (**Figure** **2**A), and fasting blood glucose increased (Figure 2C,D) as early as 8 weeks since HFD feeding, and of course, occurred after 16 weeks of HFD feeding (Figure 2E,G). Insulin tolerance did not increase significantly (Figure 2B,F). HOMA‐β decreased significantly (Figure 2H); however, HOMA‐IR of *Lgr4^pko^* decreased (Figure 2I) due to the reduction of fasting serum insulin in these mice. Glycogen contents in hepatocytes evidenced by PAS staining, were significantly decreased (Figure 2J,K). mRNA levels of hepatic genes relevant to glycolysis, such as *Gck* and *Pklr*, were significantly reduced (Figure 2L).

*Lgr4^pko^* mice showed a significant reduction in body weights (Figure S3A, Supporting Information), metabolic tissue weights (Figure S3C, Supporting Information), fat mass (Figure S3D, Supporting Information), serum cholesterol levels (Figure S3F, Supporting Information), and serum LDL‐C levels (Figure S3G, Supporting Information), though food intake remained unaltered (Figure S3B, Supporting Information). Triglyceride contents in hepatocytes evidenced by Oil Red staining, were significantly decreased (Figure S3I,J, Supporting Information). However, serum triglyceride levels increased slightly (Figure S3E, Supporting Information).

### 2.3 LGR4 might Alter both the Early Development and the After‐Development Stages of Islet β Cells

**Figure 3:**
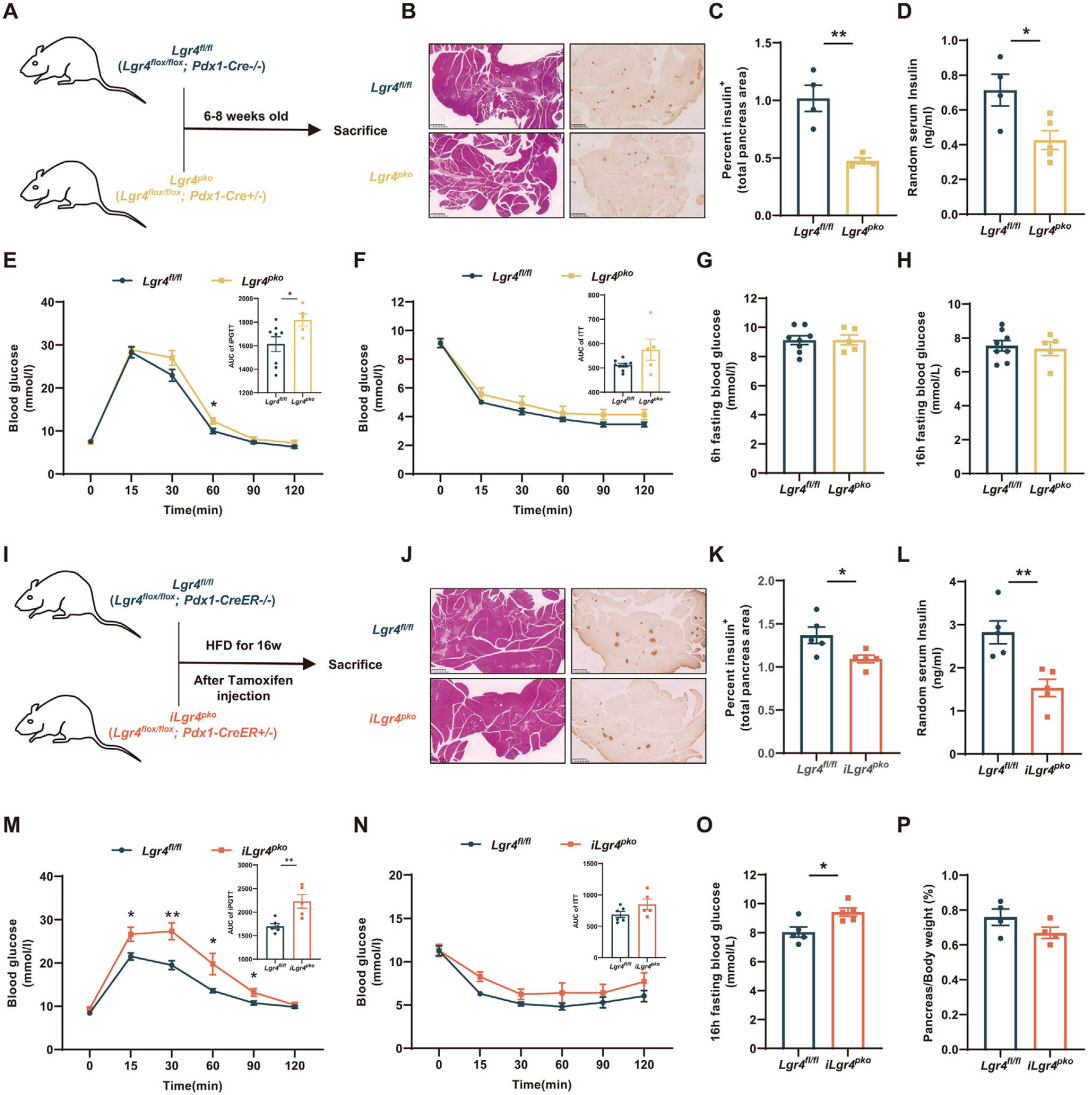
LGR4 alters both the early development and after‐development stages of islet β cells. Data are presented as mean±SEM. ^*^ *p*<0.05, ^**^ *p*<0.01 by t‐test. *Lgr4^pko^* mice and *Lgr4^fl/fl^* littermates at the age of 6–8 weeks were examined, *n* = 8 and 5 for *Lgr4^fl/fl^* and *Lgr4^pko^*. A) Schematic diagram of feeding strategy; B, C) Representative H&E, insulin immunochemistry staining, and percent insulin positive area per pancreas, *n* = 4; D) Random serum insulin levels, *n* = 4 and 5 for *Lgr4^fl/fl^* and *Lgr4^pko^*; E) IPGTT and the area under curve; F) ITT and the area under curve; G) 6 h fasting blood glucose; H) 16 h fasting blood glucose. 6–8 week‐old *iLgr4^pko^* mice and *Lgr4^fl/fl^* littermates were injected with tamoxifen for five consecutive days and fed with HFD for 16 weeks after 1 week's recovery. I) Schematic diagram of feeding strategy; J, K) Representative H&E, insulin immunochemistry staining, and percent insulin positive area per pancreas, *n* = 5; L) Random serum insulin levels, *n* = 5; M) IPGTT and the area under curve, *n* = 6 and 5 for *Lgr4^fl/fl^* and *iLgr4^pko^*; N) ITT and the area under curve, *n* = 6 and 5 for *Lgr4^fl/fl^* and *iLgr4^pko^*; O) 16 h fasting blood glucose, *n* = 5; P) Pancreas weight normalized to body weight, *n* = 4.

In 6–8 weeks‐old *Lgr4^pko^* mice, the islet area significantly decreased related to *Lgr4^fl/fl^* littermates (**Figure** **3**B,C). Consistently, these young *Lgr4^pko^* mice already had lower serum insulin levels (Figure 3D) and showed significant impairment of glucose tolerance (Figure 3E), though insulin tolerance and fasting blood glucose were not altered (Figure 3F–H). These observations indicate that LGR4 might affect early‐stage pancreas development.

To determine whether LGR4 affects the adult islet endocrine cells, we established a transgene with inducible LGR4 deficiency in adult islet β cells: *Lgr4 flox/flox, Pdx1‐CreER+/‐* (*iLgr4^pko^* mice) (Figure S1H, Supporting Information). As shown in Figure 3, LGR4 deficiency induced in adult mice reduced islet β cell mass (Figure 3J,K). Functionally, random serum insulin levels decreased (Figure 3L). In addition, impairment of glucose tolerance (Figure 3M) and an increase in fasting blood glucose levels (Figure 3O) emerged in *iLgr4^pko^* mice fed HFD for 16 weeks, while insulin tolerance did not change (Figure 3N). Pancreas weight did not change, either (Figure 3P). Generally, the reduction in islet β cell mass and impairment in glucose metabolism in *iLgr4^pko^* mice are similar to *Lgr4^pko^* mice. Our results thus indicate that LGR4 may affect both the early development and post‐development stages of islet β cells.

### 2.4 Deficiency of LGR4 in β Cells Decreases Islet Mass, Leading to More Vulnerability to Glucose Dysfunctions Induced by HFD

To further examine the effect of LGR4 on islet β cells after development, we developed another strain of inducible islet β cell *Lgr4* knock‐out mice (*Lgr4 flox/flox, Ins2‐CreER+/‐*: *iLgr4^bko^* mice) (Figure S1H, Supporting Information). *iLgr4^bko^* mice fed NCD showed a significant reduction in islet number (Figure S4B, Supporting Information) and size (Figure S4C,D, Supporting Information) compared to those of *Lgr4^fl/fl^* littermates. Functionally, *iLgr4^bko^* mice demonstrated slightly lower random serum insulin levels (Figure S4E, Supporting Information). These transgenes showed a significant impairment in glucose tolerance (Figure S4F, Supporting Information) and 6 h fasting blood glucose levels (Figure S4H, Supporting Information), and slightly higher levels of insulin tolerance (Figure S4G, Supporting Information) and 16 h fasting blood glucose (Figure S4I, Supporting Information).

**Figure 4:**
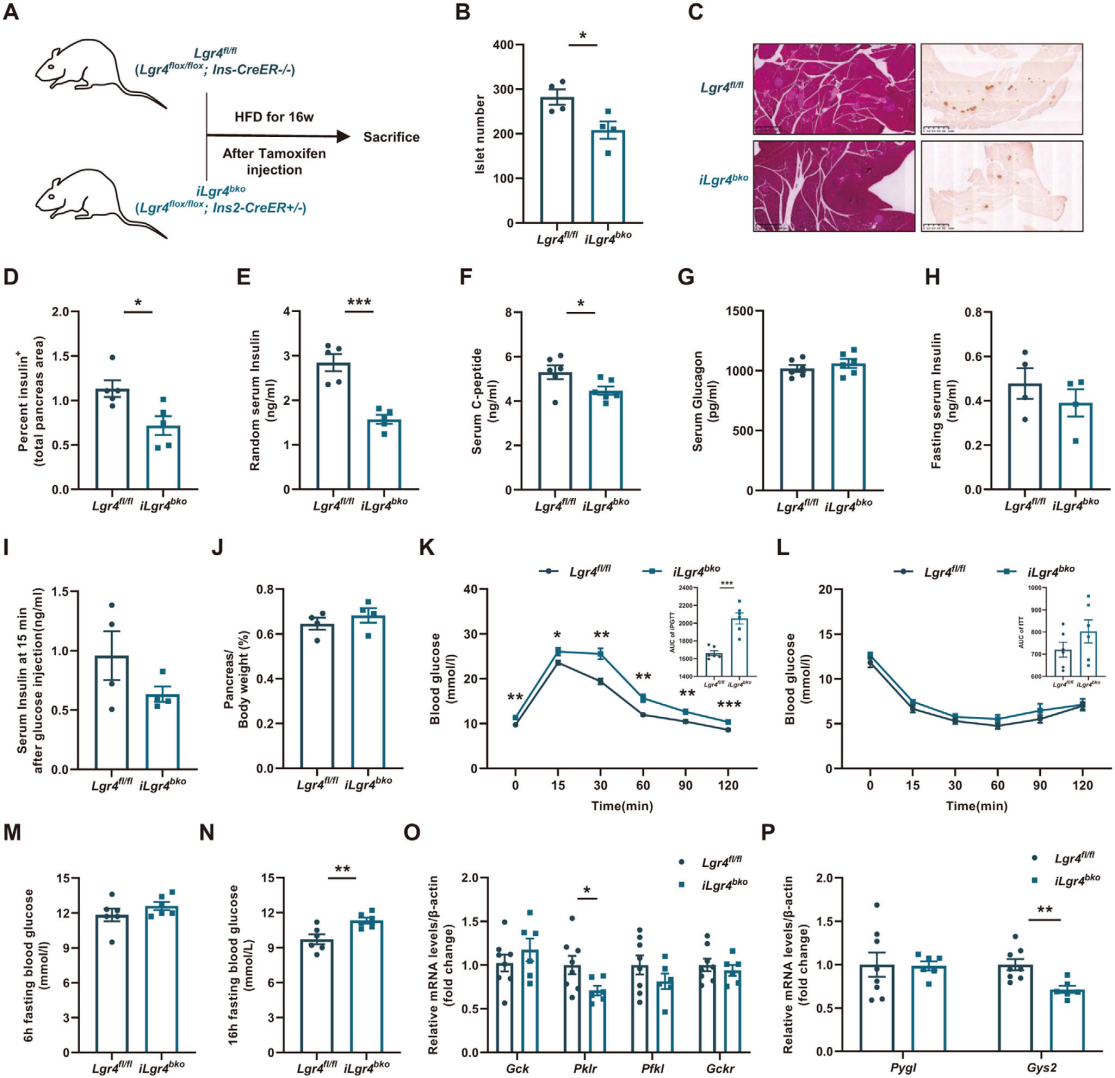
Deficiency of LGR4 decreases islet β cell mass and subsequently disrupts glucose metabolism. 6–8 week‐old *iLgr4^bko^* mice and *Lgr4^fl/fl^* littermates were injected with tamoxifen for five consecutive days and fed with HFD for 16 weeks after 1 week's recovery. Data are presented as mean±SEM. ^*^ *p*<0.05, ^**^ *p*<0.01, ^***^ *p*<0.001 by t‐test. A) Schematic diagram of feeding strategy; B) Isolated islet number, *n* = 4; C, D) Representative H&E, insulin immunochemistry staining, and percent insulin positive area per pancreas, *n* = 5; E) Random serum insulin levels, *n* = 5; F) Random serum C‐peptide levels, *n* = 6; G) Random serum glucagon levels, *n* = 6; H) 16 h fasting serum insulin levels, *n* = 4; I) Insulin levels at 15 min after glucose injection, *n* = 4; J) Pancreas weight normalized to body weight, *n* = 4; K) IPGTT and the area under curve, *n* = 6; L) ITT and the area under curve, *n* = 6; M) 6 h fasting blood glucose, *n* = 6; N) 16 h fasting blood glucose, *n* = 6; O) Liver mRNA levels of genes relevant to glycolysis, *n* = 8 and 6 for *Lgr4^fl/fl^* and *iLgr4^bko^*; P) Liver mRNA levels of genes relevant to glycogen synthesis and glycogenolysis, *n* = 8 and 6 for *Lgr4^fl/fl^* and *iLgr4^bko^*.

These alterations were more obvious in *iLgr4^bko^* mice fed with HFD for 16 weeks. Islet number (**Figure** **4**B) and β cell area (Figure 4C,D) significantly decreased. Consistently, random levels of serum insulin and C‐peptide were significantly decreased (Figure 4E,F), while serum glucagon levels, fasting serum insulin levels, and insulin levels at 15 min after being stimulated by glucose were not altered (Figure 4G–I). Pancreas weight did not change, either (Figure 4J). Associated with these alterations was a significant impairment in glucose tolerance (Figure 4K), as well as a significant increase in 16 h fasting blood glucose levels (Figure 4N), though insulin tolerance and 6 h fasting blood glucose levels did not change significantly (Figure 4L,M). Besides, mRNA levels of genes related to liver glycolysis, glycogen synthesis, and glycogenolysis (Figure 4O,P) also showed a significant alteration. To rule out the nonspecific effect of CRE recombinant enzyme activity, we fed *Ins2 CreER*+/‐ and *Ins2 CreER‐/‐* littermates with HFD for 16 weeks. No significant decrement in islet number and serum insulin levels was found between these two groups (Figure S5, Supporting Information). These results confirm that the deficiency of LGR4 in islet β cells after pancreatic development decreases islet β cell mass and function, rendering the *iLgr4^bko^* mice more vulnerable to glucose dysfunction induced by long‐term energy surplus.

### 2.5 LGR4 did not Directly Influence the Secretory Function of Islet β Cells

We next explored whether LGR4 affects the function of islet β cells. We first observed the insulin granules of *Lgr4^pko^* mice and *Lgr4^fl/fl^* littermates using a transmission electron microscope (TEM). As shown in Figure S6A (Supporting Information), *Lgr4^pko^* mice had fewer insulin granules in islet β cells relevant to *Lgr4^fl/fl^* littermates. Also, inflammation increased in the islets of these mice (Figure S6B,C, Supporting Information). However, we did not see significant changes in genes related to glucose‐sensing, insulin synthesis, and secretion in INS‐1 cells with LGR4 deficiency (Figure S6D, Supporting Information). GSIS, triglyceride, and cholesterol levels did not change in these cells (Figure S6G–I, Supporting Information). Indeed, *iLgr4^pko^* and *iLgr4^bko^* mice showed a significant impairment in glucose tolerance only in the condition of long‐term HFD feeding (16 weeks) but not in short‐term HFD feeding (8 weeks) or before HFD feeding (Figure S6J–M, Supporting Information). Also, mRNA levels of *Ins1* and *Ins2* only decreased when these transgenes were fed on HFD, but not on NCD (Figure S6N–Q, Supporting Information). All these evidences suggest that LGR4 may not directly influence the secretory function of islet β cells. Instead, LGR4 deficiency could ultimately reduce insulin levels by decreasing islet β cell mass, especially with long‐term energy surplus.

### 2.6 LGR4 Regulates β‐Cell Proliferation via Wnt‐β‐Catenin‐*Ccnd1* Pathway

**Figure 5:**
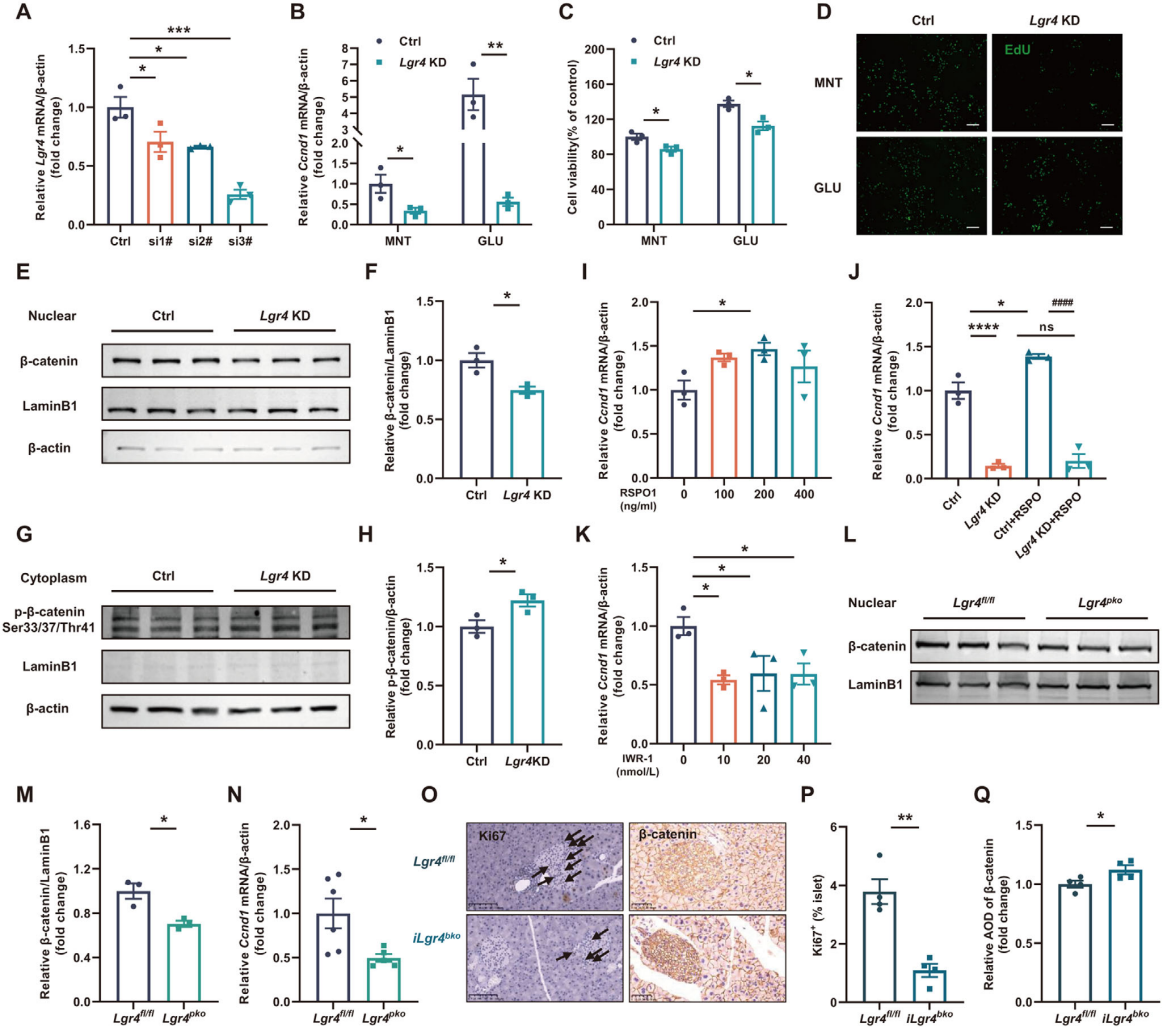
LGR4 regulates islet β cell proliferation via the Wnt‐β‐catenin‐*Ccnd1* axis. Data are presented as mean±SEM. ^*^ *p*<0.05, ^**^ *p*<0.01, ^***^ *p*<0.001, ^****^ *p*<0.0001 versus the negative control, ^####^ *p*<0.0001 versus other groups. Data in (A, I, K) were analyzed by One‐way ANOVA, data in J were analyzed by Two‐way ANOVA, and data in the other panels were analyzed by t‐test. INS‐1 cells were used to explore the mechanism by which LGR4 regulates islet β cell proliferation, *n* = 3. A) Transfection efficiency of all three strains of rat *Lgr4* siRNA. siRNA #3 was used for subsequent experiments; B–D) INS‐1 cells were transfected with *Lgr4* siRNA (*Lgr4* KD) and control siRNA (Ctrl), and treated with glucose (GLU) or mannitol control (MNT) for 48 h, relative *Ccnd1* mRNA levels (B), cell viability measured by CCK‐8 assay (C), and representative EdU staining (D); E, F) Nuclear β‐catenin levels of *Lgr4* KD and Ctrl INS‐1 cells incubated for 48 h; G, H) Cytoplasm phosphorylated β‐catenin (Ser33/37/Thr41) levels of *Lgr4* KD and Ctrl INS‐1 cells incubated for 48 h; I) *Ccnd1* mRNA levels. INS‐1 cells were treated with different concentrations of human RSPO1 recombinant protein (RSPO1) for 24 h; J) *Ccnd1* mRNA levels. *Lgr4* KD and Ctrl INS‐1 cells were treated with or without 200 ng mL^−1^ RSPO1 for 24 h; K) *Ccnd1* mRNA levels. INS‐1 cells were treated with different doses of IWR‐1 for 24 h. The mechanism was verified in transgenic mouse models. L, M) Nuclear β‐catenin levels of islets isolated from *Lgr4^pko^* mice and *Lgr4^fl/fl^* littermates fed on HFD, *n* = 3; N) *Ccnd1* mRNA levels of islets isolated from *Lgr4^pko^* mice and *Lgr4^pko^* littermates fed on HFD, *n* = 6 and 5 for *Lgr4^fl/fl^* and *Lgr4^pko^*; O–Q) Representative Ki‐67 and β‐catenin immunochemistry and quantification of *iLgr4^bko^* mice and *Lgr4^fl/fl^* littermates fed on HFD, *n* = 4.

Proliferation and apoptosis are the main factors influencing islet β cell mass. We thus first examined the effect of LGR4 signaling on the proliferation of β cells, using the rat insulinoma cell line, INS‐1 cell line. Our preliminary study had identified an *Lgr4* siRNA with high potency (**Figure** **5**A). This *Lgr4* siRNA was then used throughout the experiments. Screening genes involved in glucose sensing, islet β cell development and differentiation, insulin synthesis, insulin secretion, proliferation, and endoplasmic reticulum stress in LGR4‐deficient INS‐1 cells has identified a significant reduction of *Ccnd1* (Figure S6D, Supporting Information; Figure 5B), a gene critical for cell cycle and proliferation. CCK‐8 (Figure 5C) and EdU (Figure 5D) assays confirmed the decrease in proliferation resulting from the deficiency of LGR4, especially under the application of glucose (GLU), which could stimulate islet β cell proliferation. Rspondin (RSPO)‐LGR4 signaling has been well characterized to enhance nuclear translocation of β‐catenin, thus promoting the effect of TCF/LEF transcription factor family. Our study confirmed the decrease of β‐catenin nuclear translocation in both LGR4‐deficient INS‐1 cells and islet cells of *Lgr4^pko^* mice, and also the increase of phosphorylated β‐catenin Ser33/37/Thr41 in LGR4‐deficient INS‐1 cells (Figure 5E–H,L,M). To further confirm the effect of RSPO on *Ccnd1*, we treated the cultured INS‐1 cells with different concentrations of human RSPO1 recombinant protein. As shown in Figure 5I and Figure S6E (Supporting Information), exogenous RSPO1 significantly increased the expression of *Ccnd1*. This effect was dependent on the presence of LGR4. INS‐1 cells with a deficiency of LGR4 demonstrated no response to RSPO1 (Figure 5J). Further, inhibition of β‐catenin using IWR‐1 significantly attenuated the expression of *Ccnd1* (Figure 5K; Figure S6F, Supporting Information). Consistently, lower mRNA levels of *Ccnd1*, fewer proliferating cells, and more membrane localization of β‐catenin were detected in the islets of *Lgr4^pko^* mice and *iLgr4^bko^* mice fed with HFD for 16 weeks (Figure 5N–Q). Taken together, all these results indicate that LGR4 regulates islet β cell proliferation via the Wnt‐β‐catenin‐*Ccnd1* pathway.

### 2.7 LGR4 Suppresses Islet β Cell Apoptosis through Competing with RANKL for the Binding of RANK

**Figure 6:**
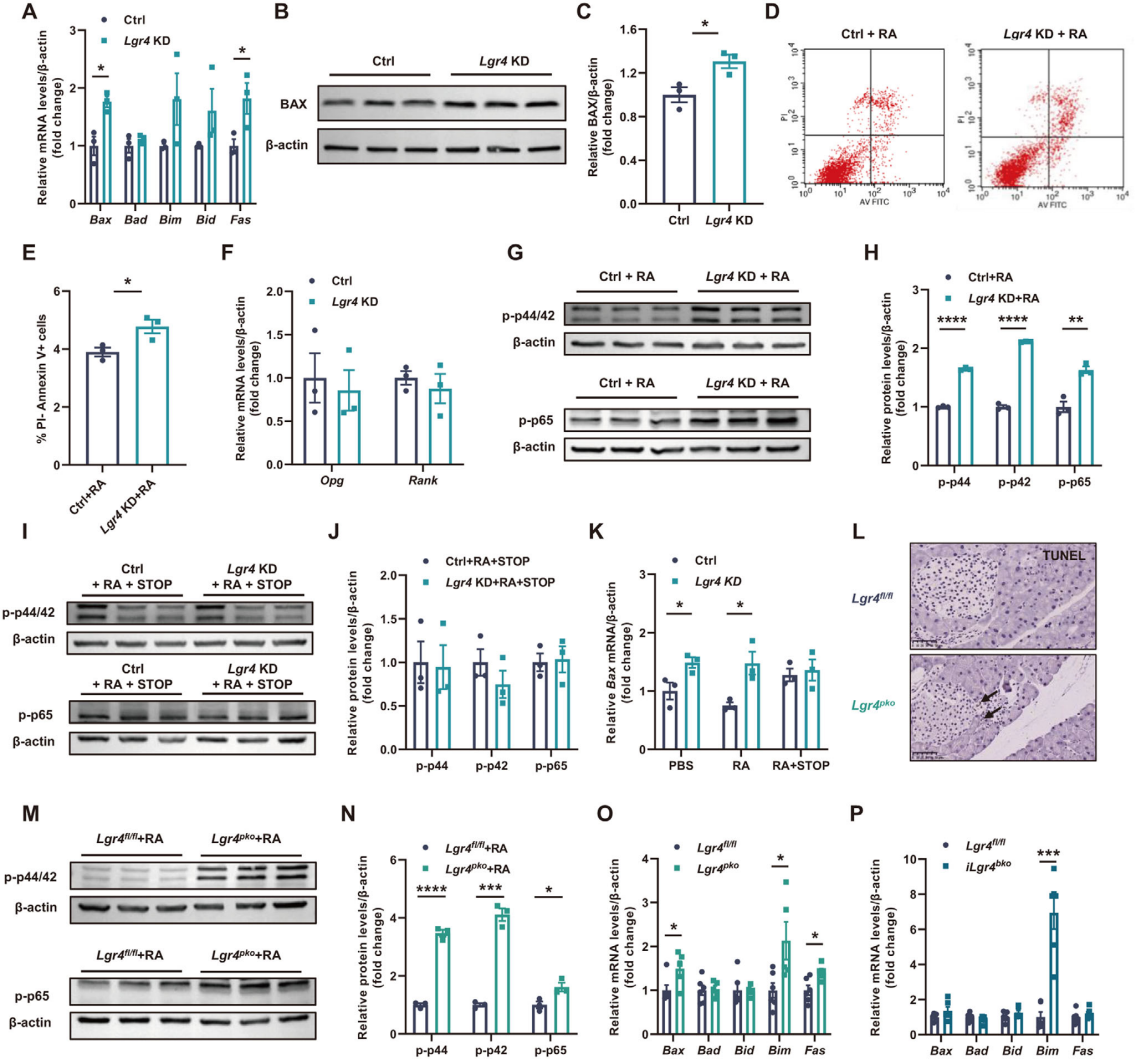
LGR4 regulates islet β cell apoptosis through competitive interaction with RANKL. Data are presented as mean±SEM. ^*^ *p*<0.05, ^**^ *p*<0.01, ^***^ *p*<0.001, ^****^ *p*<0.0001 versus the negative control by t‐test. INS‐1 cells were used to explore the mechanism by which LGR4 regulates islet β cell apoptosis, *n* = 3. A–C, F) INS‐1 cells were transfected with *Lgr4* siRNA (*Lgr4* KD) and control siRNA (Ctrl), then incubated for 48 h, relative mRNA levels of apoptosis‐related genes (A), relative protein levels of BAX (B, C), and relative mRNA levels of *Rank* and *Opg* (F); D, E, G, H) *Lgr4* KD and Ctrl INS‐1 cells were treated with 100 ng mL^−1^ RANKL (RA) for 30 min or 24 h, cells were stained with PI/Annexin‐V and detected by FCM (D, E), and levels of phosphate‐p44/p42 MAPK and phosphate‐p65 NF‐κB (G, H); I, J) *Lgr4* KD and Ctrl INS‐1 cells were co‐treated with RA and 5 µmol L^−1^ TRAF‐STOP inhibitor 6877002 (STOP), levels of phosphate‐p44/p42 MAPK and phosphate‐p65 NF‐κB; K) *Bax* mRNA levels with different treatments above. The mechanism was verified in transgenic mouse models. L) Representative TUNEL staining of islets in *Lgr4^pko^* mice and *Lgr4^fl/fl^* littermates fed on HFD, *n* = 4; M, N) Levels of phosphate‐p44/p42 MAPK and phosphate‐p65 NF‐κB. Islets were isolated from *Lgr4^pko^* mice and *Lgr4^fl/fl^* littermates and treated with 100 ng mL^−1^ RA, n = 3; O, P) Relative mRNA levels of apoptosis‐related genes of islets isolated from *Lgr4^pko^* mice, *n* = 6 and 5 for *Lgr4^fl/fl^* and *Lgr4^pko^* (O) or *iLgr4^bko^* mice, *n* = 6 (P) and their *Lgr4^fl/fl^* littermates fed on HFD.

We next examined the effect of LGR4 on the apoptosis of islet cells. Knock‐down of *Lgr4* in INS‐1 cells significantly increased the mRNA and protein levels of BAX, a key molecule associated with apoptosis (**Figure** **6**A–C). To further confirm the effects of LGR4 deficiency on islet β cell apoptosis, we used RANKL, a newly identified ligand for LGR4. PI/Annexin V staining and flow cytometry (FCM) confirmed the increase of apoptosis in LGR4‐deficient INS‐1 cells treated with RANKL recombinant protein (Figure 6D,E). Phosphorylation of p44/p42 MAPK and p65 NF‐κB, a typical alteration induced by RANKL‐RNAK signaling, was significantly increased in the absence of LGR4 and under the stimulation of RANKL (Figure 6G,H), but the expression of *Ccnd1* was not affected (Figure S7A, Supporting Information). Serum levels of RANKL didn't change (Figure S7B–E, Supporting Information), neither change were mRNA levels of *Rank* and *Opg*, the RANKL receptor and decoy receptor respectively, in both LGR4‐deficient INS‐1 cells and those transgenes (Figure 6F; Figure S7F–I, Supporting Information). These observations suggest that LGR4 deficiency activates RANKL‐RANK cascade independent of the transcription of *Rank* and *Opg*, the canonically direct effectors of RANKL‐RANK signaling. To further determine whether the effect of LGR4 deficiency on apoptosis is RANK dependent, we inhibited the interaction of RANK and downstream TNF receptor‐associated factor (TRAF) using a chemical inhibitor: TRAF‐STOP inhibitor 6877002. This inhibitor completely blocked the increase in islet β cell apoptosis induced by LGR4 deficiency (Figure 6I–K). We also confirmed the mechanism in our mouse models. In *Lgr4^pko^* mice fed HFD for 16 weeks, more apoptotic cells in islets were detected (Figure 6L). And islets isolated from these mice also had higher levels of phosphorylation of p44/p42 MAPK and p65 NF‐κB (Figure 6M,N). Also increased were the mRNA levels of apoptosis‐related genes in the islets of *Lgr4^pko^* and *iLgr4^bko^* mice (Figure 6O,P). These results indicate that LGR4 inhibits islet β cell apoptosis through decreasing the RNAKL‐RANK signaling.

We also verified our findings in human islets by analyzing samples from T2DM and non‐diabetic donors and RNA‐seq data from the database (GSE164416).^[^ ^20^ ^]^ As shown in Figure S8A,B (Supporting Information), T2DM patients had relatively lower levels of islet *LGR4*. In addition, *CCND1* expression was positively related to *LGR4* mRNA levels (Figure S8C, Supporting Information). In contrast, apoptosis‐related genes, especially *FAS*, were increased in islets from T2DM donors (Figure S8D, Supporting Information).

## 3 Discussion

Despite its abundant expression in the pancreas, whether LGR4 affects the development and function of pancreatic endocrine cells remains unclear. Our present study demonstrates that LGR4 is critical for the development and function of islet β cells. This concept is supported by the following observations: 1) Analysis of data from publicly available databases and literature revealed that islet *Lgr4* mRNA is related to metabolic disorders both in mice and human beings. 2) Using a series of transgenic mouse models with a deficiency of LGR4, we demonstrated that LGR4 is critical for islet β cell mass. This effect may involve both the early‐stage development and post‐development stage of the islet β cell. Mice with a deficiency of LGR4 in the pancreas (*Lgr4^pko^*) already showed a significant reduction in β cell mass at the age of 6–8 weeks old. Further, deficiency of LGR4 in adult islet β cells (*iLgr4^pko^* and *iLgr4^bko^*) also decreased β cell mass, especially under the circumstance of long‐term energy surplus. These observations suggest that LGR4 is essential for the maintenance of islet β cell mass after development. Although a few mouse models with *Cre* gene expression in the pancreas and islet β cells have been reported to alter physiological phenotypes,^[^ ^21^ ^]^ all three strains used in our study are not in the list. In addition, our preliminary data showed no significant decrement in the islet number and serum insulin levels between *Ins2‐CreER+/‐* and *Ins2‐CreER‐/‐* mice fed with HFD. This observation confirms that the phenotypes of all three transgenes result from the deficiency of LGR4, rather than the nonspecific effects of the CRE enzyme.

Depending on the specific ligands, LGR4 may activate different signaling pathways to exercise distinct physiological functions.^[^ ^22^ ^]^ For example, binding of LGR4 with Rspondin potentiates Wnt‐β‐catenin signaling.^[^ ^6^, ^7^, ^8^ ^]^ The β‐catenin signaling is critical for cell proliferation.^[^ ^23^ ^]^ Consistently, deficiency of LGR4 in the pancreas significantly decreased islet β cell proliferation measured by Ki67, EdU, and CCK8 assay. On the other hand, activation of LGR4 by Rspondin significantly increased the expression of *Ccnd1*, a cell cycle‐related gene, and β‐cell proliferation. This effect was attenuated by the β‐catenin inhibitor IWR‐1, suggesting a β‐catenin‐dependent mechanism. Since the proliferation of islet β cells is time‐consuming in adult islets,^[^ ^24^ ^]^ the effect of LGR4 deficiency on β cell mass in adult mice may not be solely attributed to cell proliferation. Consistent with previous reports demonstrating that apoptosis plays an important role in the maintenance of β cell mass, our study demonstrates that deficiency of LGR4 decreased β cell mass by increasing apoptosis. Both in vivo and in vitro data showed that deficiency of LGR4 increased apoptosis in the islet cells. RANKL, which traditionally binds with RANK to promote cell apoptosis,^[^ ^25^ ^]^ significantly increased the phosphorylation of p44/p42 MAPK and p65 NF‐κB in the absence of LGR4. Inhibiting the interaction of RANK and downstream TRAF by TRAF‐STOP inhibitor 6877002 eliminated the up‐regulation of β cell apoptosis induced by LGR4 deficiency. Taken together, our data demonstrate that LGR4 suppresses islet β cell apoptosis via interfering with the interaction of RANKL and RANK.

Though we mainly focused on proliferation and apoptosis, we also investigated the effect of LGR4 on insulin biosynthesis and secretion. Interestingly, deficiency of insulin biosynthesis and secretion could only be observed in those transgenes fed on HFD, but not on NCD. Also, knockdown of LGR4 didn't change the expression of *Ins2* or GSIS in INS‐1 cells. These data suggest that LGR4 does not directly affect the biosynthesis and secretion of insulin, but the reduction of islet β cell number and long‐time challenge of excessive energy supply could result in decompensation of islet β cell function, thus affecting insulin production and secretion.

Three strains of transgenes were applied in this research, and they differ from each other according to the time when the CRE recombinase begins to function effectively. Briefly, *Pdx1‐Cre* causes a deletion of the targeted gene as early as embryonic day 9.0 in multipotent progenitor (MP) cells, and thus in all kinds of cells derived from MP cells in later stages. Therefore, we could not rule out the possibility that there are alterations in the exocrine portions of *Lgr4^pko^* mice. Also, we propose that LGR4 might affect early stages of development (including embryonic and/or postnatal stages) since *Lgr4^pko^* mice showed a decrease of islet β cell area and function as early as 6–8 weeks old, when they were just reaching adulthood. More evidence is needed to prove this hypothesis. While Tamoxifen was injected to *iLgr4^pko^* and *iLgr4^bko^* mice when they were adult, resulting in a deletion of the targeted gene in mature islet β cells. Data collected from these transgenes together indicate that LGR4 at least plays a role in mature islet β cells, by affecting the proliferation and apoptosis.

Lipid metabolism of *Lgr4^pko^* mice fed with HFD seemed counterintuitive, because serum triglyceride increased while body weight, serum cholesterol, serum HDL‐C, and liver triglyceride decreased. The possible reasons of this counterintuitive phenotype might be: 1) LGR4 deficiency accelerated the process of T2DM, and *Lgr4^pko^* mice on HFD were in a state like late‐stage T2DM. In this stage, circulating insulin significantly decreased, glucose intake was inhibited, and the catabolism of lipid and protein was enhanced; therefore, these transgenic mice were more emaciated. Also, liver function was abnormal, resulting in lower levels of apolipoprotein synthesis. 2) LGR4 might also influence the function of acinar cells and/or ductal cells, while the exocrine portion of the pancreas also plays a significant role in food digestion and absorption. The decrease in body weight, serum cholesterol, serum HDL‐C, and liver triglyceride might indicate that these transgenic mice were relatively malnourished. In our future work, we will explore whether and how LGR4 plays a role in the exocrine portion of the pancreas.

In summary, our work demonstrates a physiological function of LGR4 in the islet β cells. Deficiency of LGR4 in the pancreas and islet β cells impairs the flexibility of islet β cells upon the challenge of long‐term energy surplus. This occurs through the suppression of RSPO‐LGR4‐β‐catenin‐*Ccnd1* signaling and concurrent increase of RANKL‐RANK signaling, leading to a subsequent decrease of proliferation and increase of apoptosis, respectively. Our study thus reveals LGR4 as a potential target to promote islet β cell flexibility for the intervention of type 2 diabetes.

## 4 Experimental Section

### Human Pancreatic Tissue Sections

Paraffin‐embedded human pancreas tissue sections used in this study were obtained from non‐diabetic and T2DM donors at Shanghai Changzheng Hospital with informed consent of the deceased organ donor's families (Ethical Approval: 2018SL004F). All the research protocols used in this study were in strict compliance with all relevant legal and ethical regulations of Peking University (Ethical Approval: IRB00001052‐25034).

### Animals and Treatment

This study exclusively examined male mice because mainly focus was on the phenotypes upon the challenge of a high‐fat diet (HFD), while the body weights of female mice showed a huge variability under this condition. All experiments were conducted in strict accordance with the Guide for the Care and Use of Laboratory Animals prepared by the National Academy of Sciences (NIH publication 86‐23, revised 1985). Animal experimental protocols were approved by the Animal Care and Use Committee of Peking University (BCJB0035). *Pdx1‐Cre* mice (B6. FVB‐Tg (Pdx1‐cre) 6Tuv/J, Stock No: 014647, from The Jackson Laboratory) were bred with *Lgr4 flox/flox* mice (from Helmholtz Zentrum, Germany)^[^ ^15^ ^]^ to generate pancreas *Lgr4* knock‐out mice (*Lgr4 flox/flox, Pdx1‐Cre+/‐*). *Ins2‐CreER* mice (STOCK Tg (Ins2‐cre/ERT) 1Dam/J, Stock No: 008122, from The Jackson Laboratory) and *Pdx1‐CreER* mice (STOCK Tg (Pdx1‐cre/Esr1*) #Dam/J, Stock No: 024968, from The Jackson Laboratory) were bred with *Lgr4 flox/flox* mice to generate islet β cell *Lgr4* knock‐out mice (*Lgr4 flox/flox, Ins2‐CreER+/‐* and *Lgr4 flox/flox, Pdx1‐CreER+/‐*) after five consecutive days of tamoxifen induction. Six to eight‐week‐old conditional knock‐out mice and their littermate control (*Lgr4 flox/flox, Cre ‐/‐*: WT mice) were fed with a 10% kcal normal chow diet (NCD) or a 60% kcal high‐fat diet (HFD) for 16 weeks. Animals were housed in a standard environment (22±2 °C, humidity at 50 ± 15%) with a 12‐h light and 12‐h dark cycle. Food and water were freely accessible except for the fasting experiments. Animals and food were weighed at the same time every week.

### Materials

Rabbit anti‐LGR4 (20150‐1‐AP), mouse anti‐β‐actin (66009‐1‐Ig) from Proteintech, rabbit anti‐phospho‐β‐catenin (Ser33/37/Thr41) (9561), rabbit anti‐β‐catenin (8480), rabbit anti‐Bax (2772), rabbit anti‐p44/p42 MAPK (4695), rabbit anti‐phospho‐p44/p42 MAPK (4370) from Cell Signaling Technology, rabbit anti‐NF‐κB p65 (A19653), rabbit anti‐phospho‐NF‐κB p65 (AP1294), rabbit anti‐Bax (A19684) from ABclonal were used for Western blot analysis. Rabbit anti‐LGR4 (PA5‐109908) from Invitrogen, rabbit anti‐F4/80 (70076) and rabbit anti‐Insulin (4590) from Cell Signaling Technology, and rabbit anti‐Ki67 (ab16667) from Abcam were used for immunochemistry analysis. Rabbit anti‐Insulin (PTM‐5642) from PTM BIO, mouse anti‐Glucagon (G2654) from Sigma‐Aldrich, and rabbit anti‐Somatostatin (ab30788) from Abcam were used for immunofluorescence analysis.

Rat *Lgr4* siRNAs were synthesized by Generay Biotechnology (Shanghai, China). RNAi in vitro transfection reagent (DN001‐10) was obtained from D‐Nano Therapeutics (Beijing, China). IWR‐1 (HY‐12238) and TRAF‐STOP inhibitor 6877002 (HY‐110247) were obtained from MedChem Express (Monmouth Junction, NJ, USA). Rat RANKL recombinant protein (APA855Ra61) was purchased from CLOUD‐CLONE (Wuhan, China), and human RSPO1 recombinant protein (CX83) was obtained from Novoprotein (Suzhou, China).

### Tissue Sample Preparation and Histological Analysis

Animals were sacrificed at the end of the experiment after 4–6 h of fasting to standardize their feeding rhythm. Tissue samples, including serum, pancreas, liver, subcutaneous white adipose tissue (sWAT), epididymal white adipose tissue (eWAT), and brown adipose tissue (BAT), were harvested for further analysis. For the pancreas, the whole tissues were harvested and fixed with 4% paraformaldehyde in PBS for 24 h, and then embedded in paraffin and sectioned into 4 µm‐thick slices at an interval of 150 µm. Three consecutive slices at each interval were stained for the insulin immunoreactivity, and the average percentage of insulin‐positive (islet β cell) area over the whole pancreas was calculated using ImageJ. For other tissues, parts of the tissues were extracted and fixed with 4% paraformaldehyde in PBS for 24 h, and then embedded in paraffin and sectioned into 4 µm‐thick slices for the subsequent analysis. All histological analyses were performed according to the manufacturer's instructions.

### Intraperitoneal Glucose Tolerance Test (IPGTT) and Insulin Tolerance Test (ITT)

For IPGTT, mice were fasted for 16 h in a clean environment, and then intraperitoneally injected with glucose at a dose of 2 or 1.5 g kg^−1^ for mice fed with NCD or HFD, respectively. Blood was collected from the tip of the tail at 0, 15, 30, 60, 90, and 120 min after injection, and the glucose concentration was immediately measured by a glucometer (Acuu‐Chek Active, Roche, Germany). For ITT, mice were fasted for 6 h in a clean environment, and then intraperitoneally injected with insulin at a dose of 0.75 or 1 U kg^−1^ for mice fed with NCD or HFD, respectively. Blood was collected from the tip of the tail at 0, 15, 30, 60, 90, and 120 min after injection, and the glucose concentration was immediately measured by a glucometer.

### Isolation of Islets

Mice were euthanized, and HBSS containing 0.5 mg mL^−1^ collagenase P was injected into the common bile duct of the mice, and then the pancreas was extracted and digested for 17 min at 37 °C. Enzyme digestion was stopped by HBSS containing 5% FBS, and the tissues were shaken vigorously to fully dissociate. Islets were collected under the stereo microscope, and pictures were immediately taken for the calculation of islet number and size. Harvested islets were cultured in RPMI‐1640 supplemented with 10% FBS and 1% penicillin/streptomycin for 24 h for subsequent experiments.

For Western blot analysis of phospho‐p44/p42 MAPK and phospho‐p65, ≈200 islets were fasted for 2 h and then treated with 100 ng mL^−1^ RANKL for 30 min before harvest.

### Glucose Stimulated Insulin Secretion (GSIS)

Islets were transferred into RPMI‐1640 with no FBS to starve for 12 h. Thirty islets were incubated in 1 mL KRB buffer solution with no glucose for 1 h to balance, and then in 1 mL KRB buffer solution with 2.8 mmol L^−1^ glucose (low glucose) for 1 h, and finally in 1 mL KRB buffer solution with 25 mmol L^−1^ glucose (high glucose) for 1 h. Supernatants were collected at the end of every step of incubation, and the insulin concentrations were measured with a commercial ELISA kit. The secreted insulin was normalized to the protein content of the islets.

### Cell Culture

The rat insulinoma cell line, INS‐1 cells (RRID: CVCL_0352), were obtained from the Cell Resource Center, IBMS, CAMS/PUMC, and were detected to be free of mycoplasma. INS‐1 cells were cultured in a humid atmosphere (5% CO_2_) at 37 °C using RPMI‐1640 culture medium supplemented with 10% heat‐inactivated FBS, 1 mmol L^−1^ sodium pyruvate, 50 µmol L^−1^ β‐Mercaptoethanol, and 1% penicillin/streptomycin. Cells were cultured in a 6‐well or 12‐well plate and grown until ≈70% confluency before transfection. siRNA transfection was conducted under the manufacturer's instructions. Cells were harvested for q‐PCR and Western blot analyses 48 h after transfection. RSPO1, IWR‐1, RANKL, and TRAF‐STOP inhibitor 6877002 were applied 24 h after transfection, and cells were cultured for another 24 h before harvest. But for Western blot analysis of phospho‐p44/p42 MAPK and phospho‐p65, RANKL was used 30 min before harvest, and TRAF‐STOP inhibitor 6877002 was used 30 min before the usage of RANKL, 48 h after transfection.

### EdU Staining

INS‐1 cells were transfected with siRNA and then cultured in the above culture medium supplemented with an extra 19.5 mmol L^−1^ glucose (to a final concentration of 25 mmol L^−1^ glucose) or 19.5 mmol L^−1^ mannitol as a control to balance the osmotic pressure for 48 h. The cells were incubated with 20 mmol L^−1^ EdU for 2 h, and then harvested for analysis.

### Statistics

Prism software was used for graphing and statistical analysis, and the experimental results were shown as mean ± SEM. Statistical significance of differences between two groups and three or more groups (considering only one variable) was analyzed with Student t‐test and One‐way ANOVA, respectively. Statistical significance of differences considering two variables was analyzed with Two‐way ANOVA. *p*<0.05 denotes statistical significance.

### Data Availability

The raw data supporting the conclusions of this article will be made available by the authors without undue reservation.

## Conflict of Interest

The authors declare no conflict of interest.

## Author Contributions

C.L. and Y.F. contributed equally to this work. W.Z. and C.L. designed the experiment. C.L. accomplished all the experiments, bred the animals, analyzed the results, and drafted the manuscript. H.Y. and Y.F. collected and analyzed human samples. J.M. helped with islet isolation and GSIS analysis. Y.Z. helped with animal breeding. Z.Z. and L.S. provided technical support. J.M. provided expertise. Y.Y. and W.Z. obtained funding, analyzed the results, and revised the manuscript. All authors edited and approved the final manuscript.

## Supporting information



Supporting Information

## Data Availability

The data that support the findings of this study are available in the maintext and/or Supporting information of this article. Raw data supporting the findings of this study are available from the corresponding authors upon reasonable request.
