# Supplementary material for: Deficiency of Leucine‐Rich Repeat Containing G Protein‐Coupled Receptor 4 in Pancreas Reduces β Cell Mass
Source: Adv Sci (Weinh). 2025 Aug 14;12(41):e08858. doi: 10.1002/advs.202508858 (PMC12591167; doi:10.1002/advs.202508858)
Supplement: Supplementary file 1 — Supporting Information [file ADVS-12-e08858-s001.docx]

**Title:** Deficiency of leucine-rich repeat containing G protein-coupled receptor 4 in pancreas reduces β cell mass

**Authors:** Chao Luo^1#^, Yifan Feng^3#^, Jiajie Min^1^, Yan Zhao^1^, Ziming Zhu^1^, Lijun Sun^1^, Hao Yin^3^*, Yue Yin^2^*, and Weizhen Zhang^1^*

**Affiliations:**

1. Department of Physiology and Pathophysiology, School of Basic Medical Sciences, State Key Laboratory of Vascular Homeostasis and Remodeling, Peking University, Beijing, 100191, China.

2. Department of Pharmacology, School of Basic Medical Sciences, State Key Laboratory of Vascular Homeostasis and Remodeling, Peking University, Beijing, 100191, China.

3. Organ Transplant Center, Shanghai Changzheng Hospital, Second Affiliated Hospital of Naval Medical University, Shanghai, 200003, China.

#These authors contributed equally: Chao Luo, Yifan Feng

***Correspondence:**

Weizhen Zhang

Department of Physiology and Pathophysiology, School of Basic Medical Sciences, State Key Laboratory of Vascular Homeostasis and Remodeling, Peking University, Beijing, 100191, China.

E-mail: weizhenzhang@bjmu.edu.cn

or

Yue Yin

Department of Pharmacology, School of Basic Medical Sciences, State Key Laboratory of Vascular Homeostasis and Remodeling, Peking University, Beijing, 100191, China.

E-mail: yueyin@bjmu.edu.cn

or

Hao Yin

Organ Transplant Center, Shanghai Changzheng Hospital, Second Affiliated Hospital of Naval Medical University, Shanghai 200003, China.

E-mail: yinhao@czh-tx.com

**Conflict-of-interest statement:**

The authors have declared that no conflict of interest exists.

**Supplementary Data**

**Table S1. Mutations of human *LGR4* and the related metabolic disorder traits**

| Risk Allele | P Value | Risk Frequencyncy | Mapped Genes | Trait Name |
| --- | --- | --- | --- | --- |
| rs4536164-A | 6.00E-12 | 0.3137 | LGR4 | Waist-hip ratio |
| rs4514364-? | 4.00E-18 | NR | LGR4 | Body mass index |
| rs4128868-? | 3.00E-09 | NR | LGR4 | Body mass index |
| rs12804815-G | 5.00E-06 | 0.98 | LGR4 | Urate levels in obese individuals |
| rs11825412-T | 5.00E-11 | NR | LGR4 | Body mass index (MTAG) |
| rs11030003-C | 1.00E-08 | NR | LGR4 | Childhood body mass index |
| rs11030003-C | 7.00E-06 | NR | LGR4 | BMI (weighted GWA) |
| rs11030003-C | 2.00E-13 | NR | LGR4 | BMI (standard GWA) |
| rs7952482-C | 5.00E-14 | NR | LGR4 | Body mass index |
| rs35070613-A | 9.00E-06 | 0.9048 | LGR4-AS1 | Weight |
| rs1038101-G | 9.00E-11 | NR | LGR4, CCDC34 | Body mass index |
| rs142981556-? | 6.00E-09 | NR | LIN7C, LGR4-AS1 | Body mass index |
| rs12786130-C | 6.00E-10 | 0.238203 | LGR4-AS1, LIN7C | HDL cholesterol levels |
| rs12786130-C | 3.00E-10 | 0.238203 | LGR4-AS1, LIN7C | Cholesteryl ester levels in HDL |
| rs4922787-G | 4.00E-09 | 0.238409 | LGR4-AS1, LIN7C | Cholesterol levels in large HDL |
| rs4922787-G | 2.00E-09 | 0.238409 | LGR4-AS1, LIN7C | Cholesteryl ester levels in large HDL |
| rs12786130-C | 1.00E-08 | 0.238203 | LGR4-AS1, LIN7C | Concentration of large HDL particles |
| rs11030026-A | 4.00E-10 | 0.163176 | LGR4-AS1, LIN7C | Cholesterol to total lipids in large LDL percentage (UKB data field 23615) |
|  |  |  |  |  |

Information from GWAS Database (https://www.ebi.ac.uk/gwas/genes/LGR4) shows that at least 13 mutations in human *LGR4* gene locus are related to metabolic disorders.

**
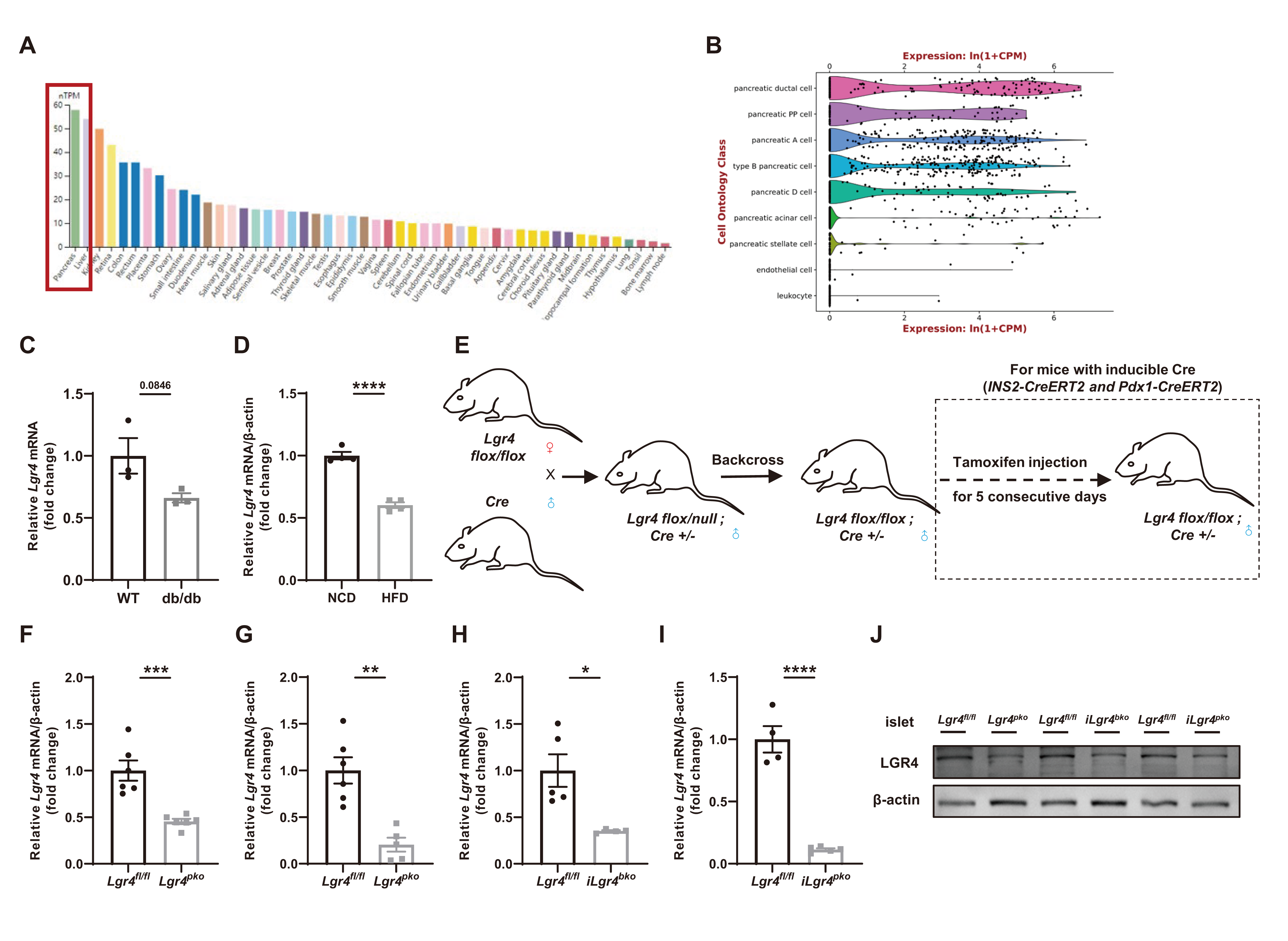
**

**Figure S1. Expression features of LGR4, its relevance to metabolic disorders and strategy of transgenic mice breeding**

Data are presented as mean±SEM. *p<0.05, **p<0.01, ***p<0.001, ****p<0.0001 by t-test.

(A) Expression of *LGR4* in different human organs, and the figure was derived from The Human Protein Atlas (https://www.proteinatlas.org/LGR4); (B) Expression of *Lgr4* in different cell types of mouse pancreas, and the figure was derived from Tabula Muris (https://tabula-muris.ds.czbiohub.org).

(C) Expression of islet *Lgr4* in wild type (WT) and db/db mice, the data was derived from GSE107489; (D) WT mice were fed with NCD or HFD for 16 weeks, islet *Lgr4* mRNA levels.

(E) Schematic diagram of transgenic mice breeding; (F-I) *Lgr4* mRNA levels of pancreas from *Lgr4^pko^* mice and *Lgr4^fl/fl^* littermates, n=6 (F), islets from *Lgr4^pko^* mice and *Lgr4^fl/fl^* littermates, n=6 and 5 for *Lgr4^fl/fl^* and *Lgr4^pko^* (G), islets from *iLgr4^bko^* mice and *Lgr4^fl/fl^* littermates, n=5 and 4 for *Lgr4^fl/fl^* and *iLgr4^bko^* (H), islets from *iLgr4^pko^* mice and *Lgr4^fl/fl^* littermates, n=4 and 5 for *Lgr4^fl/fl^* and *iLgr4^pko^* (I); (J) Representative LGR4 protein levels of islets from *Lgr4^pko^*, *iLgr4^bko^*, *iLgr4^pko^* and their *Lgr4^fl/fl^* littermates.

**
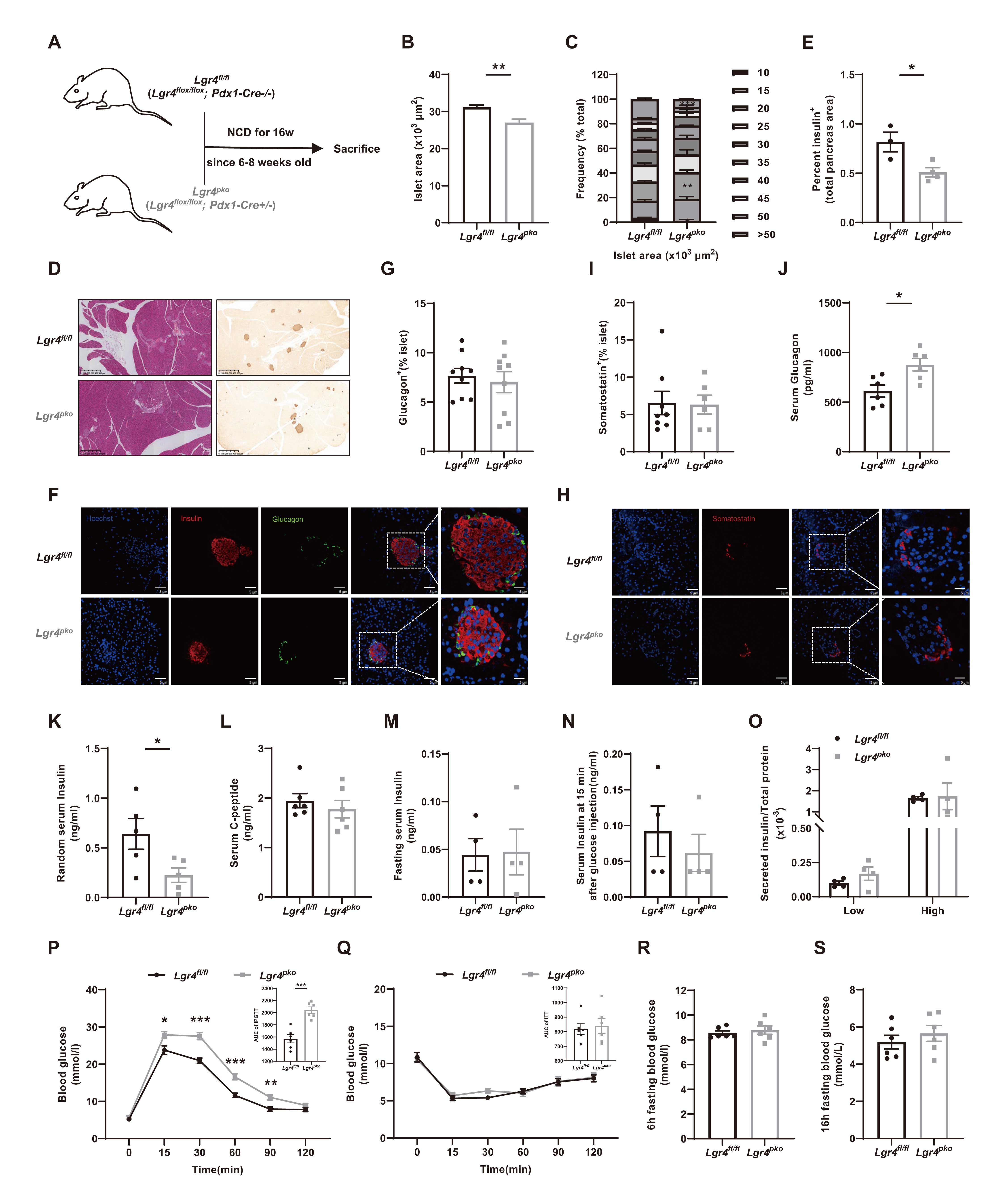
**

**Figure S2. Deficiency of pancreas LGR4 decreases islet β cell mass and function on NCD**

Six to eight-week-old *Lgr4^pko^* mice and *Lgr4^fl/fl^* littermates were fed with NCD for 16 weeks. Data are presented as mean±SEM. *p<0.05, **p<0.01 by t-test.

(A) Schematic diagram of feeding strategy; (B, C) Average islet area and distribution of islet area. n=953 and 230 islets isolated from *Lgr4^fl/fl^* and *Lgr4^pko^* mice respectively; (D, E) Representative H&E, insulin immunochemistry staining, and percent insulin positive area per pancreas, n=3 and 4 for *Lgr4^fl/fl^* and *Lgr4^pko^*; (F, G) Representative insulin (Red) and glucagon (Green) immunofluorescence staining, and percentage of glucagon positive area per islet, n=9; (H, I) Representative somatostatin (Red) immunofluorescence staining, and percentage of somatostatin positive area per islet, n=8 and 6 for *Lgr4^fl/fl^* and *Lgr4^pko^*; (J) Random serum glucagon levels, n=6; (K) Random serum insulin levels, n=5; (L) Random serum C-peptide levels, n=4; (M) Sixteen hour fasting serum insulin levels, n=6; (N) Insulin levels at 15 minutes after glucose injection, n=4; (O) GSIS measured at low (2.8 mmol/L) and high (25 mmol/L) glucose levels, n=4; (P) IPGTT and the area under curve, n=6; (Q) ITT and the area under curve, n=6; (R) Six hour fasting blood glucose, n=6; (S) Sixteen hour fasting blood glucose, n=6.

**
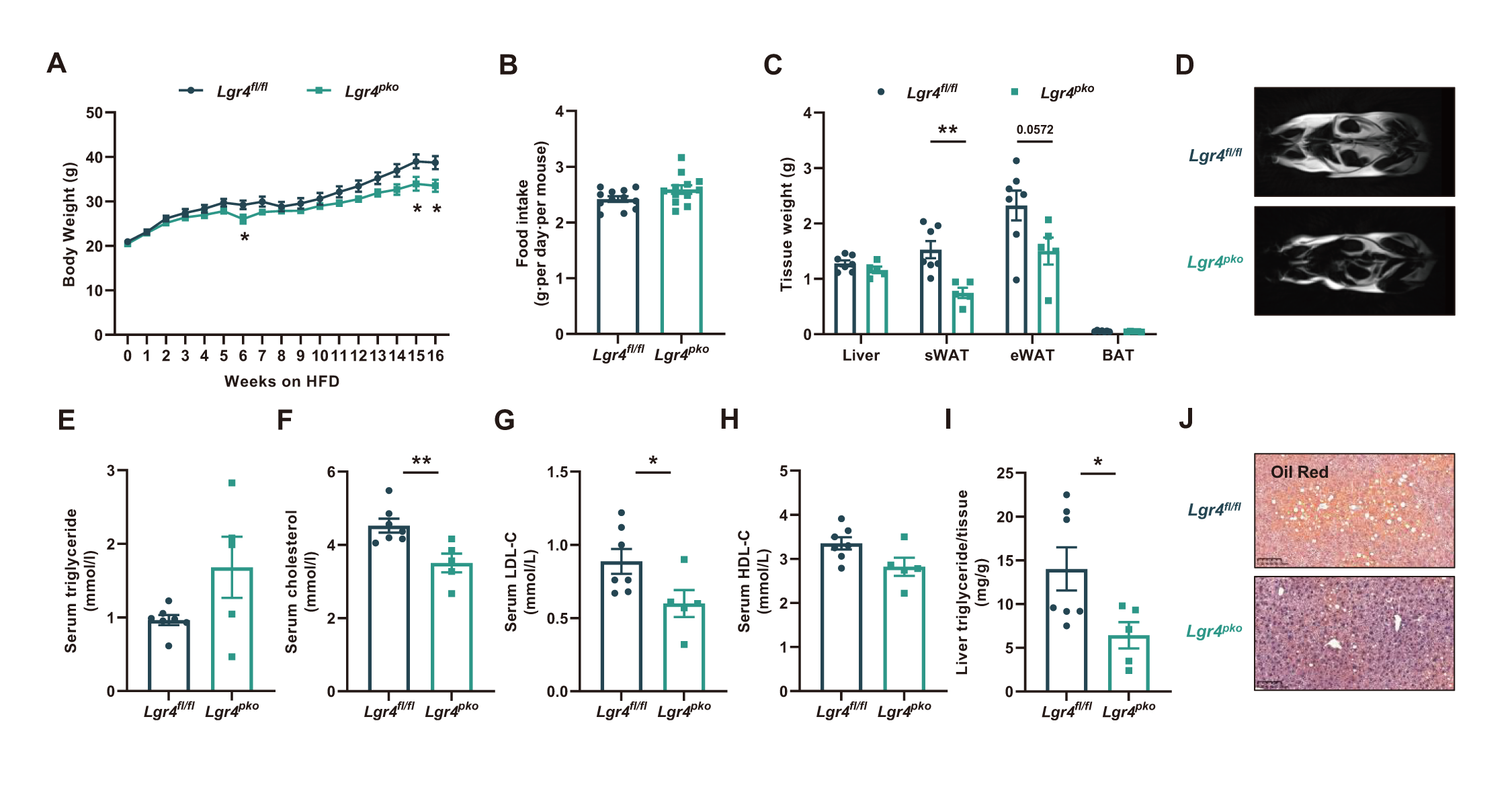
**

**Figure S3. *Lgr4^pko^* mice on HFD suffered from abnormal lipid metabolism**

Six to eight-week-old *Lgr4^pko^* mice and *Lgr4^fl/fl^* littermates were fed with HFD for 16 weeks. Data are presented as mean±SEM. *p<0.05, **p<0.01 by t-test. n=7 and 5 for *Lgr4^fl/fl^* and *Lgr4^pko^.*

(A) Body weight of every week; (B) Average food intake per day per mouse, n=12 weeks; (C) Metabolic tissue weights; (D) Representative MRI screening images, the white part is fat mass; (E) Serum triglyceride levels; (F) Serum cholesterol levels; (G) Serum LDL-C levels; (H) Serum HDL-C levels; (I) Content of liver triglyceride, normalized to the extracted tissue weight; (J) Representative Oil Red staining of liver.


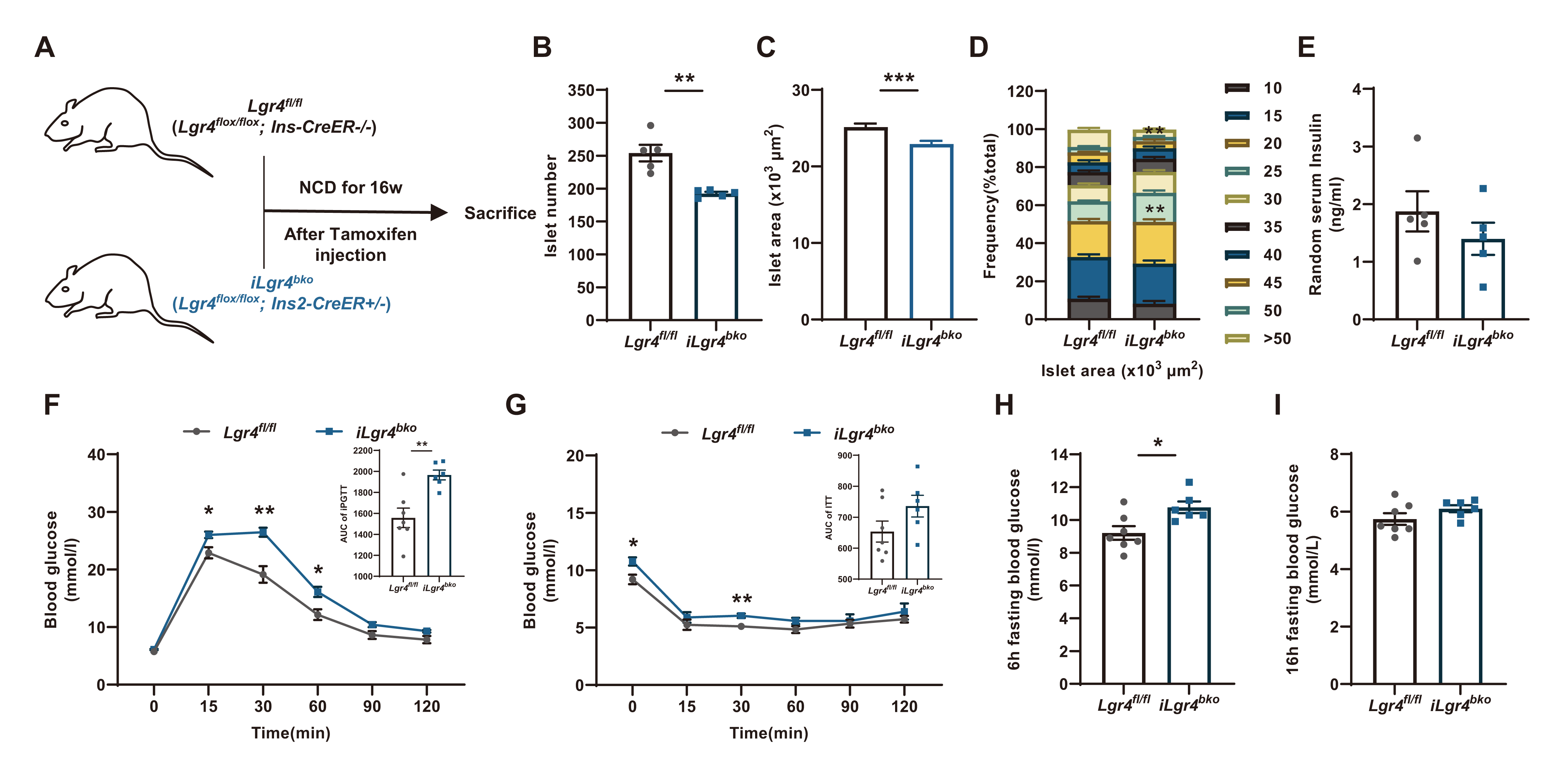


**Figure S4. Deficiency of LGR4 decreases islet β cell mass on NCD**

Six to eight-week-old *iLgr4^bko^* mice and *Lgr4^fl/fl^* littermates were injected with tamoxifen for five consecutive days and fed with NCD for 16 weeks one week later. Data are presented as mean±SEM. *p<0.05, **p<0.01, ***p<0.001 by t-test.

(A) Schematic diagram of feeding strategy; (B) Isolated islet number, n=5; (C, D) Average islet area and distribution of islet area. n=1270 and 963 islets isolated from *Lgr4^fl/fl^* and *iLgr4^bko^* mice respectively; (E) Random serum insulin levels, n=5; (F) IPGTT and the area under curve, n=6 and 5 for *Lgr4^fl/fl^* and *iLgr4^bko^*; (G) ITT and the area under curve, n=6 and 5 for *Lgr4^fl/fl^* and *iLgr4^bko^*; (H) Six hour fasting blood glucose levels, n=6 and 5 for *Lgr4^fl/fl^* and *iLgr4^bko^*; (I) Sixteen hour fasting blood glucose levels, n=6 and 5 for *Lgr4^fl/fl^* and *iLgr4^bko^*.

**
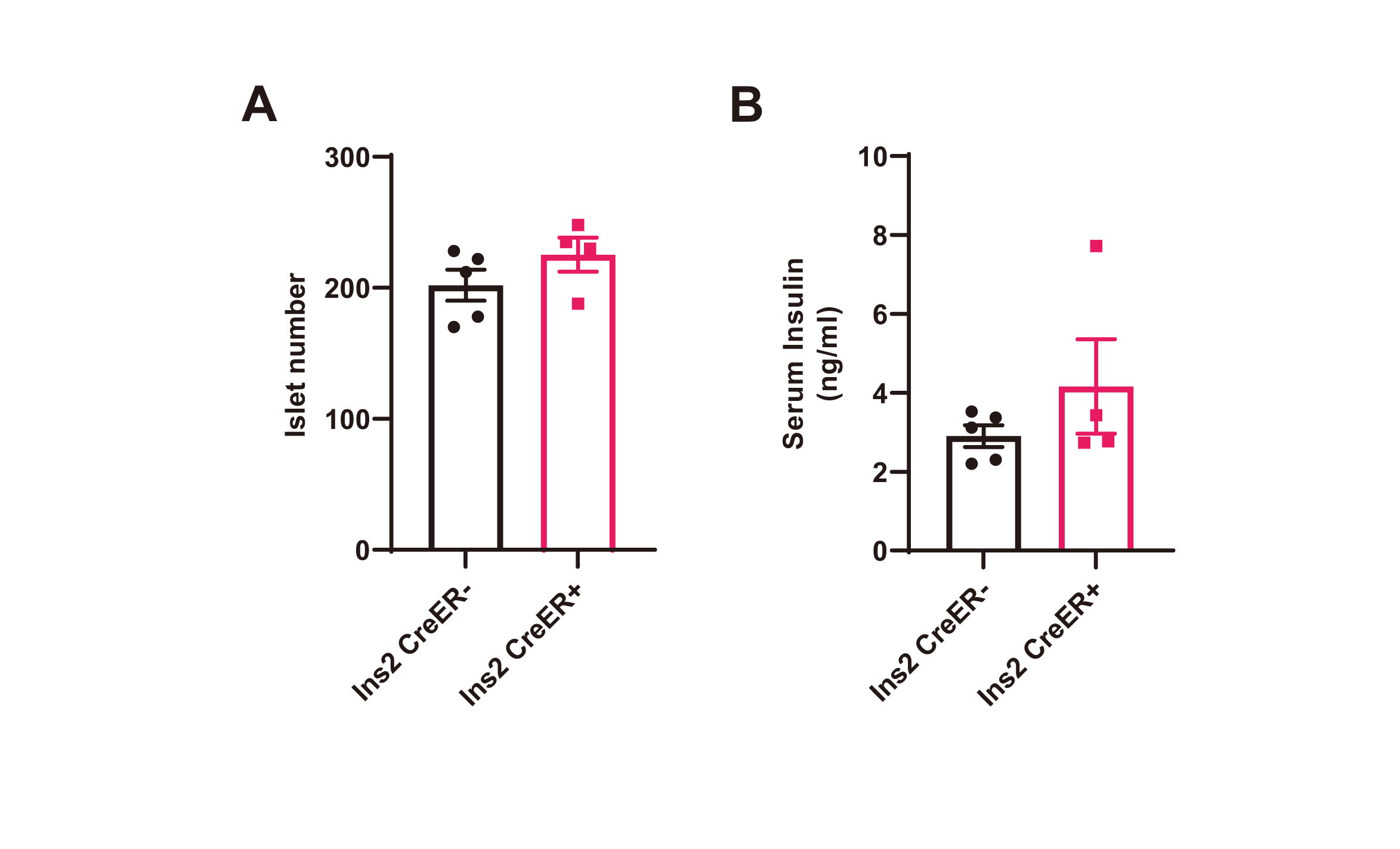
**

**Figure S5. The expression of *Cre* does not decrease islet numbers and insulin levels**

Six to eight-week-old *Ins2 CreER+/-* and *Ins2 CreER-/-* littermates were injected with tamoxifen for five consecutive days and fed with HFD for 16 weeks one week later, n=5 and 4 for *Ins2 CreER-/-* and *Ins2 CreER+/-*. (A) Islet number; (B) Random serum insulin levels.

**
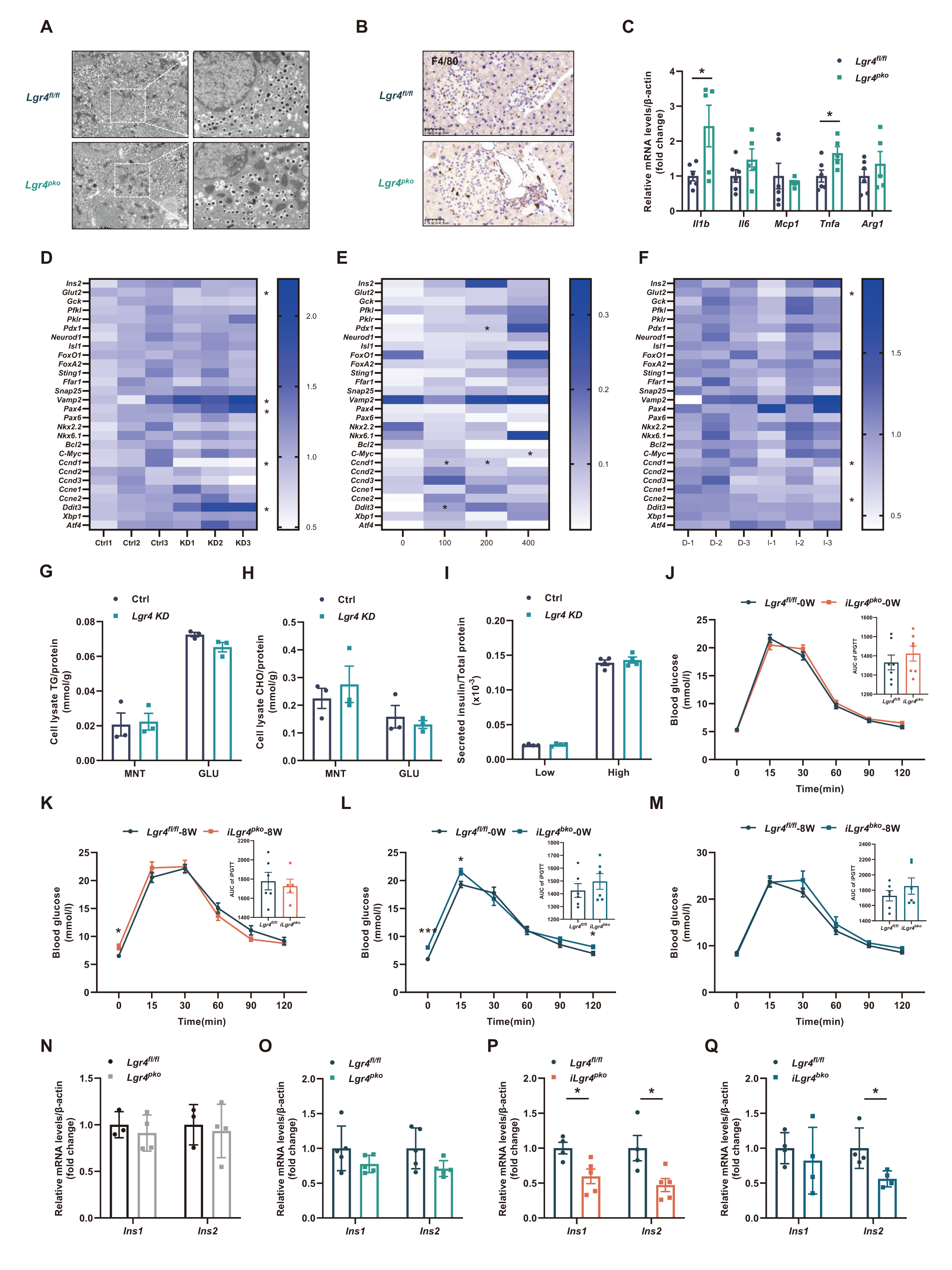
**

**Figure S6. Deficiency of LGR4 does not directly disrupt the secretion of insulin**

Data are presented as mean±SEM. *p<0.05 by t-test.

(A-C) *Lgr4^pko^* mice and *Lgr4^fl/fl^* littermates were fed on HFD for 16 weeks, representative transmission electron microscope (TEM) images of insulin granules in islet β cells (A), representative F4/80 immunochemistry staining of islets (B), and islet mRNA levels of genes relevant to inflammation, n=6 and 5 for *Lgr4^fl/fl^* and *Lgr4^pko^.*

(D) Gene expression profile of INS-1 cells transfected with *Lgr4* siRNA (*Lgr4* KD) and control siRNA (Ctrl) and incubated for 48 hours; (E) Gene expression profile of INS-1 cells treated with different concentrations of RSPO1 for 24 hours; (F) Gene expression profile of INS-1 cells treated with 10 μmol/L IWR-1 for 24 hours. (G) Levels of triglyceride in the cell lysate; (H) Levels of cholesterol in the cell lysate; (I) GSIS of *Lgr4* KD and Ctrl INS-1 cells. n=3.

(J, K) IPGTT and the area under curve when *iLgr4^pko^* mice and *Lgr4^fl/fl^* littermates before they were fed on HFD (HFD for 0 week), n=7 (J), and when they were fed on HFD for 8 weeks, n=6 and 5 for *Lgr4^fl/fl^* and *iLgr4^pko^* (K); (L, M) IPGTT and the area under curve when *iLgr4^bko^* mice and *Lgr4^fl/fl^* littermates before they were fed on HFD (HFD for 0 week), n=6 (L), and when they were fed on HFD for 8 weeks, n=6 (M).

(N-Q) mRNA levels of *Ins1* and *Ins2* in islets derived from *Lgr4^pko^* mice on NCD, n=3 and 4 for *Lgr4^fl/fl^* and *Lgr4^pko^* (N), *Lgr4^pko^* mice on HFD, n=5 (O), *iLgr4^pko^* mice on HFD, n=4 and 5 for *Lgr4^fl/fl^* and *iLgr4^pko^* (P) and *iLgr4^bko^* mice on HFD, n=4 (Q) and *Lgr4^fl/fl^* littermates.


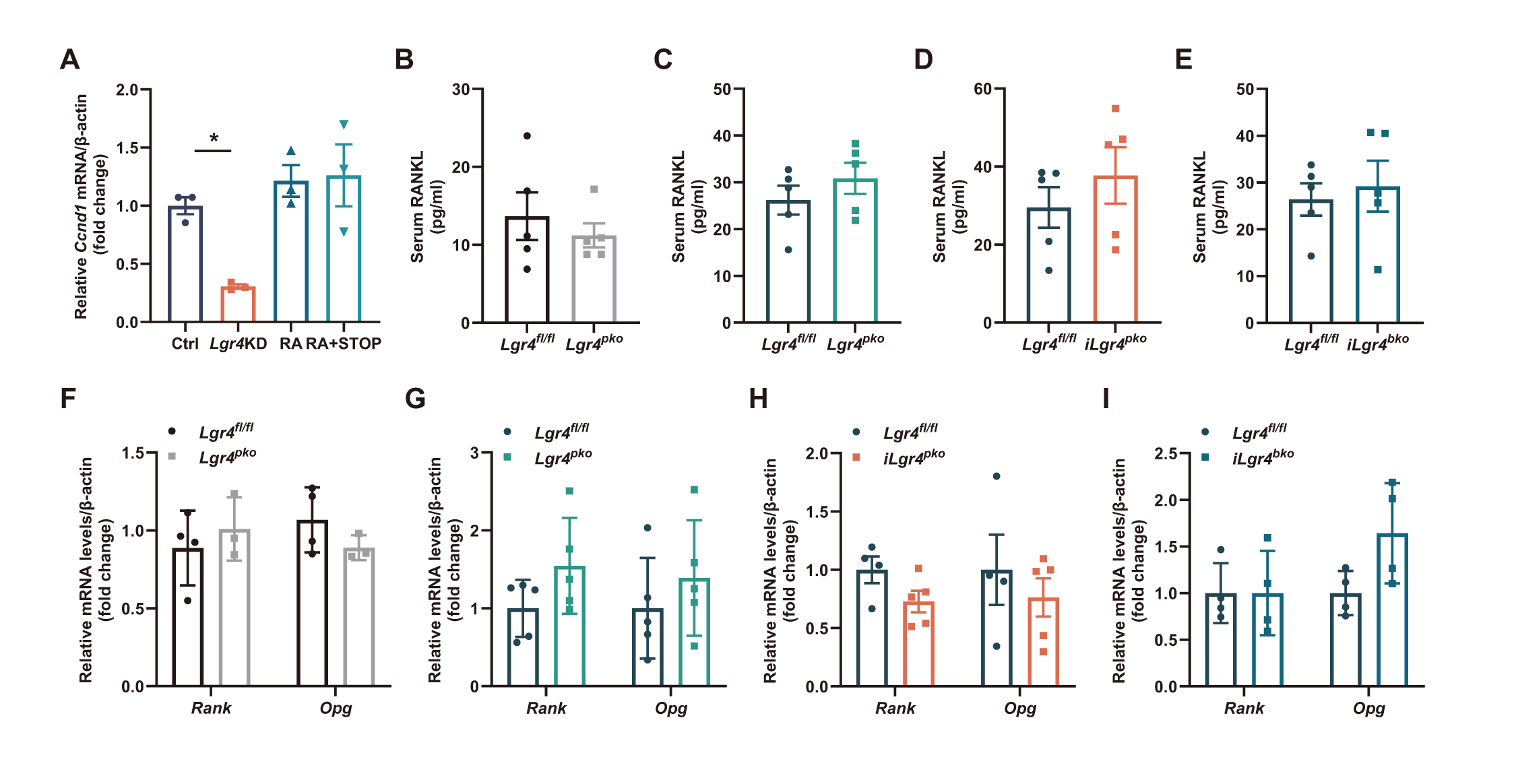


**Figure S7. Deficiency of LGR4 does not alter the content of RANKL and expression of RANK**

Data are presented as mean±SEM. *p<0.05 by t-test.

(A) *Lgr4* KD and Ctrl INS-1 cells were treated with RA or co-treated with RA and STOP for 24 hours, the mRNA levels of *Ccnd1*, n=3.

(B-E) Serum RANKL levels of *Lgr4^pko^* mice on NCD, n=3 and 4 for *Lgr4^fl/fl^* and *Lgr4^pko^* (B), *Lgr4^pko^* mice on HFD, n=5 (C), *iLgr4^pko^* mice on HFD, n=4 and 5 for *Lgr4^fl/fl^* and *iLgr4^pko^* (D) and *iLgr4^bko^* mice on HFD, n=4 (E) and *Lgr4^fl/fl^* littermates.

(F-I) mRNA levels of *Rank* and *Opg* in islets derived from *Lgr4^pko^* mice on NCD, n=3 and 4 for *Lgr4^fl/fl^* and *Lgr4^pko^* (F), *Lgr4^pko^* mice on HFD, n=5 (G), *iLgr4^pko^* mice on HFD, n=4 and 5 for *Lgr4^fl/fl^* and *iLgr4^pko^* (H) and *iLgr4^bko^* mice on HFD, n=4 (I) and *Lgr4^fl/fl^* littermates.


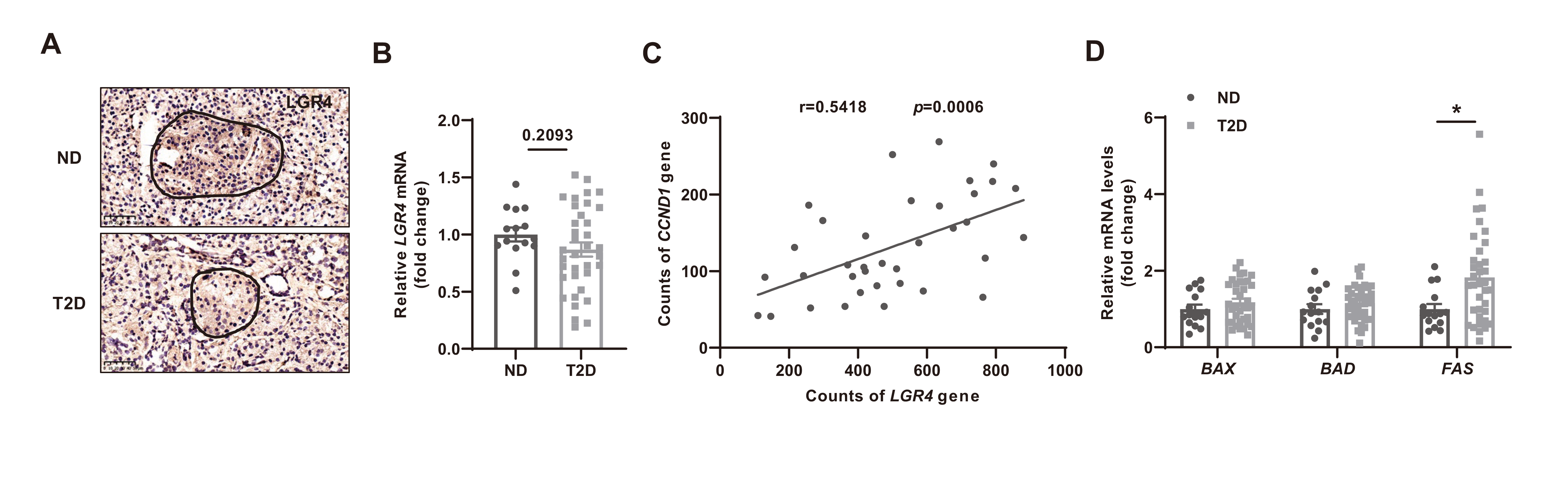


**Figure S8. LGR4 and downstream signals are relevant to T2DM in human beings**

Data are presented as mean±SEM. *p<0.05 by t-test.

(A) Representative LGR4 immunochemistry staining of human islets in T2DM and non-diabetic donors. n=4 and 5 for ND and T2D donors.

(B-D) Data from GSE164416 were reanalyzed. 15 ND and 36 T2D samples were adopted (eliminating those with significantly abnormal *LGR4, CCND1, BAX, BAD* and *FAS* expression). The expression of *LGR4* in two groups (B), the correlation of *LGR4* and *CCND1* in all samples (C), and the expression of genes relevant to apoptosis (D).

**Supplementary information**

Table 1. siRNA used in this work

| Strain name | Sense (5'-3') | Antisense (3'-5') |
| --- | --- | --- |
| Rat-*Lgr4#1* | ACUGAUAAGCAUAUGGUACUG | GUACCAUAUGCUUAUCAGUGU |
| Rat-*Lgr4#2* | UAAGUUAGACACAGAAAUCAA | GAUUUCUGUGUCUAACUUACU |
| Rat-*Lgr4#3* | AAGUGUUUGCAUGAUACUGGU | CAGUAUCAUGCAAACACUUAA |

Table 2. Primers for Quantitative RT-PCR (Mouse)

| Genes | Upstream primer | Downstream primer |
| --- | --- | --- |
| *Actb* | ATCTGGCACCACACCTTC | AGCCAGGTCCAGACGCA |
| *Lgr4* | AACCTCATTTCCTACGGAAGGC | GATTGGCAAAGTCTCTGGCTG |
| *Pparg* | TCAGCTCTGTGGACCTCTCC | ACCCTTGCATCCTTCACAAG |
| *Acaca* | TGGTCGTGACTGCTCTGTGC | GTAGCCGAGGGTTCAGTTCC |
| *Fasn* | TGGGTTCTAGCCAGCAGAGT | ACCACCAGAGACCGTTATGC |
| *Dgat2* | CGTGACGTGCATTGGCTTC | TGGAGGGCTGAGAGGATGC |
| *Gpam* | CACACGAGCAGGAAAGATGA | GGACTGCATAGATGCTGCAA |
| *Scd1* | GCGATACACTCTGGTGCTCA | CCCAGGGAAACCAGGATATT |
| *Gys2* | ACCAAGGCCAAAACGACAG | GGGCTCACATTGTTCTACTTGA |
| *Pygl* | GAGAAGCGACGGCAGATCAG | CTTGACCAGAGTGAAGTGCAG |
| *Gck* | ATGGCTGTGGATACTACAAGGA | TTCAGGCCACGGTCCATCT |
| *Pfkl* | GTGTCTGCCCTGGCTTCC | CCTCTGCGATGATGATGATGT |
| *Pklr* | ACATCATCTTTGCCTCCTTTG | TCACGCCTTCATGGTTCTC |
| *Gckr* | CCAAGCACCAAGCGGTATCA | GTCAGTGGGTTGGACTTCTCT |

Table 3. Primers for Quantitative RT-PCR (INS-1 cell line, Rat)

| Genes | Upstream primer | Downstream primer |
| --- | --- | --- |
| *Lgr4* | TTCAGTTTCCATCAGCAGCCAAGG | CAGCAGTCACAGACAGTCAGGTTAC |
| *Glut2* | ACACCAGCACATACGACACCAG | AAGAACGAGGCGACCATTCCG |
| *Gck* | AGCAGAAGGGAACAACATCGTAGG | GGCGGTCTTCATAGTAGCAGGAG |
| *Pfkl* | TGAATCTGCGGCTGATGCTGAAG | CTCAAGGTGCGGCGTGTGAC |
| *Pklr* | TACCACCGCCAGTTGTTTGAGG | ACGGCTCCAATCGCAGTGAC |
| *Pdx1* | TGCCAGAGTTCAGTGCTAATCCC | CTTCCCTGTTCCAGCGTTCCC |
| *Neurod1* | GGACAGACGAGTGCCTCAGTTC | TTCCTCCTCCTCTTCCTCCTCTTC |
| *Isl1* | TCCACAAACAGCCCGAGAAGAC | CGTAGCAGGTCCGCAAGGTG |
| *FoxO1* | GTACGCCGACCTCATCACCAAG | GTAGGGCACACTCTTCACCATCC |
| *FoxA2* | GGACACAGGCTTCTCAGGTTCAG | CAGAGGCAGGTGTTCCCTTCAG |
| *String1* | AGTTGTGCCATGTCCAGTCCAG | ACCAGGATGCCAAGATGCCAAG |
| *Ffar1* | GGGACTGGCTCTTGGCTTGG | CCACGCTTCCAGGCAGACC |
| *Snap25* | CCTGCCCGTGTGGTGGATG | GATGCCGCTCACCTGCTCTAG |
| *Vamp2* | TGCACCTCCTCCAAATCTTACCAG | ACCTTGTCCACATTCACCCTCATG |
| *Pax4* | CCAGCCACAGGAATCGGACTATC | GCTTTCCACGGACAACTGAATCTG |
| *Pax6* | ACCAGTGTCTACCAGCCAATTCC | TGGGCATCGGCGGCAAAG |
| *Nkx2.2* | GCCAGCCTCATCCGTCTCAC | GGCGTCACCTCCATACCTTTCTC |
| *Nkx6.1* | CGCACGGCATCAACGACATC | ACGAGGAGGAGGAGGAAGAAGAC |
| *Bcl2* | ACGAGGAGGAGGAGGAAGAAGAC | GGTTGCTCTCAGGCTGGAAGG |
| *Bax* | GACGCATCCACCAAGAAGCTGAG | GCTGCCACACGGAAGAAGACC |
| *Bad* | CGGGACAGGCAGCCAATAACAG | TCCTCCATCCCTTCATCTTCCTCAG |
| *Bim* | TGCGGCGGATCGGAGACG | CCAGACCAGACGGAAGATGAATCG |
| *Bid* | CTCGCCCAAGCAGGTGATGAAC | GCCTTGTCATTCTCCATGTCCCTAG |
| *C-myc* | TCTATCACCAGCAACAGCAGAGC | GCAACATAGGACGGAGAGCAGAG |
| *Ccnd1* | GCTACCGCACAACGCACTTTC | CGCAGGCTTGACTCCAGAAGG |
| *Ccnd2* | ACGACTTCATTGAGCACATCCTACG | TGGCGGGTACATGGCAAACTTG |
| *Ccnd3* | GGACCTGGCTGCTGTGATTGC | TGGCGATCATGGATGGAGGGTAC |
| *Ccne1* | CAACATCCAGACCCACACCAACAG | CCTGCTCACTGCTCTGCTTCTTAC |
| *Ccne2* | GCCGCCGCTGCCTTATGC | GGCACCATCCAGTCTACACATTCTG |
| *Ddit3* | CTCGCTCTCCAGATTCCAGTCAG | TGTGCCACTTTCCTCTCATTCTCC |
| *Xbp1* | TCTCAGAGGCAGAGTCCAAGGG | AGAGGCAACAGCGTCAGAATCC |
| *Atf4* | ATGACCGAGATGAGCTTCCTGAAC | CCGCCTTGTCGCTGGAGAAC |
| *Opg* | TGTGTCCCTTGCCCTGACTACTC | CTCGGTTGTGGGTGCGGTTG |
| *Rank* | CAAGGGACGACGGAATCAGATGTG | TGAAGAGGAGCAGGACGATGAGAC |
